# Structural and biochemical rationale for enhanced spike protein fitness in delta and kappa SARS-CoV-2 variants

**DOI:** 10.1038/s41467-022-28324-6

**Published:** 2022-02-08

**Authors:** James W. Saville, Dhiraj Mannar, Xing Zhu, Shanti S. Srivastava, Alison M. Berezuk, Jean-Philippe Demers, Steven Zhou, Katharine S. Tuttle, Inna Sekirov, Andrew Kim, Wei Li, Dimiter S. Dimitrov, Sriram Subramaniam

**Affiliations:** 1 Department of Biochemistry and Molecular Biology, University of British Columbia, Vancouver, BC V6T 1Z3 Canada; 2 BC Centre for Disease Control, Vancouver, BC V5Z 4R4 Canada; 3 Center for Antibody Therapeutics, Division of Infectious Diseases, Department of Medicine, University of Pittsburgh School of Medicine, 3550 Terrace Str, Pittsburgh, PA 15261 USA

**Keywords:** Electron microscopy, Cryoelectron microscopy

## Abstract

The Delta and Kappa variants of SARS-CoV-2 co-emerged in India in late 2020, with the Delta variant underlying the resurgence of COVID-19, even in countries with high vaccination rates. In this study, we assess structural and biochemical aspects of viral fitness for these two variants using cryo-electron microscopy (cryo-EM), ACE2-binding and antibody neutralization analyses. Both variants demonstrate escape of antibodies targeting the N-terminal domain, an important immune hotspot for neutralizing epitopes. Compared to wild-type and Kappa lineages, Delta variant spike proteins show modest increase in ACE2 affinity, likely due to enhanced electrostatic complementarity at the RBD-ACE2 interface, which we characterize by cryo-EM. Unexpectedly, Kappa variant spike trimers form a structural head-to-head dimer-of-trimers assembly, which we demonstrate is a result of the E484Q mutation and with unknown biological implications. The combination of increased antibody escape and enhanced ACE2 binding provides an explanation, in part, for the rapid global dominance of the Delta variant.

## Abstract (web summary)

Saville, Mannar *et al*. provide a structural basis for enhanced antibody evasion and ACE2 binding by the Delta SARS-CoV-2 spike protein. They further identify a head-to-head dimer-of-trimers cryoEM reconstruction unique to the Kappa variant spike.

## Introduction

**Fig. 1:**
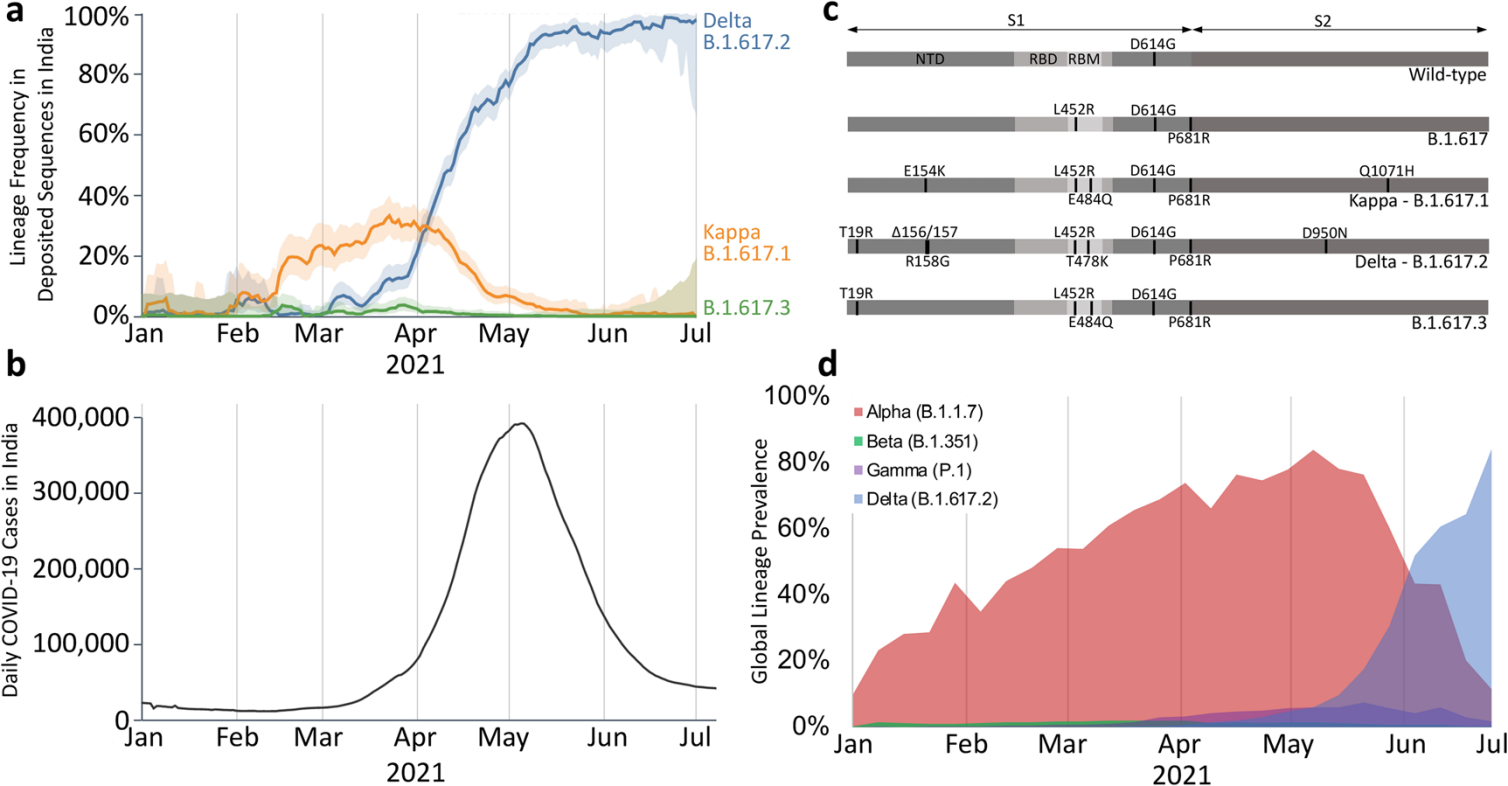
Emergence and prevalence of the Delta and Kappa SARS-CoV-2 lineages. **a** Delta (B.1.617.2), Kappa (B.1.617.1) and B.1.617.3 lineage frequency in India from January to July 2021. Deposited sequences were downloaded from the GISAID initiative. The 95% confidence interval is plotted as the lighter shaded border around each line. This figure panel was generated using the outbreak.info project^3^. **b** Daily confirmed COVID19 cases in India as curated by the outbreak.info project^3^. **c** SARS-CoV-2 amino acid sequence box plots for the wild-type (D614G) and B.1.617 sub-lineages. The abbreviations are NTD N-terminal domain, RBD receptor-binding domain, RBM receptor-binding motif. **d** Global prevalence for the Alpha, Beta, Gamma and Delta lineages of SARS-CoV-2 from January to July 2021. The sequence data were downloaded from the GISAID initiative and plotted as the percentage of the global prevalence for each week^1,2^.

In March 2021, genomic sequencing of SARS-CoV-2 samples in Maharashtra, India revealed an increased prevalence of E484Q, L452R and P681R co-mutation in the Spike glycoprotein (S protein)^1–3^. This variant was called the double mutant by the global news media and was later designated as lineage B.1.617.1 and then the Kappa variant of interest by the World Health Organization^4^. The Kappa (B.1.617.1) lineage is a sub-lineage of the B.1.617 lineage, which is defined by L452R and P681R co-mutation. By early April 2021, the Kappa variant accounted for ~35% of all sequenced cases in India, which coincided with the start of a rise in daily COVID-19 cases (Fig. 1a, b)^1–3^. In addition to the B.1.617 and B.1.617.1 variants, variants B.1.617.2 and B.1.617.3 saw increased prevalence in March 2021. While the B.1.617 and B.1.617.3 variants never exceeded 5% of sequenced cases in India, the B.1.617.2 variant (now designated as the Delta variant of concern) rapidly became dominant in India within months. Given the precursory identification of the Kappa variant, it was initially thought to be the major contributor to the increased COVID-19 case numbers in India. However, retrospective analyses show the rapid dominance of the Delta variant to better coincide with the exponential surge of COVID-19 cases in India (Fig. 1a, b).

Following the emergence of the Delta variant in India, it rapidly spread globally and in early June 2021, it overtook the Alpha (B.1.1.7) variant to become the major SARS-CoV-2 lineage worldwide (Fig. 1d)^1–3^. The Kappa variant, despite harbouring multiple mutations in the S protein that have been demonstrated to increase viral fitness, has not significantly spread globally, with only a few travel-linked clusters reported^5–7^. The exact mutations present within the Kappa and Delta S proteins have been reported variously, suggesting a degree of variability in their classification^8–12^. Within the receptor-binding domain (RBD) of the S protein, both the Kappa and Delta variants share an identical substitutional mutation (L452R) with the previously emerged variants of interest B.1.427/429 (Epsilon)^1,2,4,8^. The S protein E484Q mutation in the Kappa variant was similarly found in the Beta (B.1.351) and Gamma (P.1) lineages, where residue 484 is mutated to lysine (E484K)^1,2^. The Delta variant contains a novel T478K mutation within the RBD - that is not present in previous variants of concern - with uncharacterised effect. After the RBD, the NTD is the second highest-targeted S protein domain by SARS-CoV-2 convalescent sera antibodies^13^. The NTD is comprised of five loops (called N1-5; N1: residues 14–26, N2: residues 67–79, N3: residues 141–156, N4: residues 177–189 and N5: residues 246–260), with N1, N3 and N5 loops dubbed an NTD neutralisation supersite for the propensity of neutralising antibodies to target this location^14^. While the Kappa variant contains only a single mutation (E154K) in N3, the Delta variant contains a mutation in N1 (T19R) and multiple mutations in N3 (Δ156/157, R158G). Finally, the P681R mutation (present in all B.1.617 lineages) immediately precedes the furin cleavage consensus sequence (682-RRSR/SVA-688), with initial reports suggesting that the P681R mutation enhances cleavage, and consequently S protein post-fusion transition^11,15,16^. How the combinatorial effect of the Delta and Kappa S protein mutations contribute towards increased viral fitness remains superficially characterised relative to previously emerged variants of concern (Alpha, Beta and Gamma).

Herein, we report cryo-electron microscopy (cryo-EM) structures of Delta and Kappa variant S protein trimers – both in the unbound state and in complex with the ACE2 receptor – to gain insight into how their mutations underlie changes in ACE2 binding and antibody neutralisation escape. Through our experiments, we uncovered an unexpected dimerisation phenomenon for the Kappa variant S protein with – as yet – unknown biological implications. We go on to report two additional structures of S proteins containing novel substitutions at position 484 (I484 and A484) to dissect the chemical nature of this dimerisation event.

## Results

### Antibody evasion by Delta and Kappa spike proteins

**Fig. 2:**
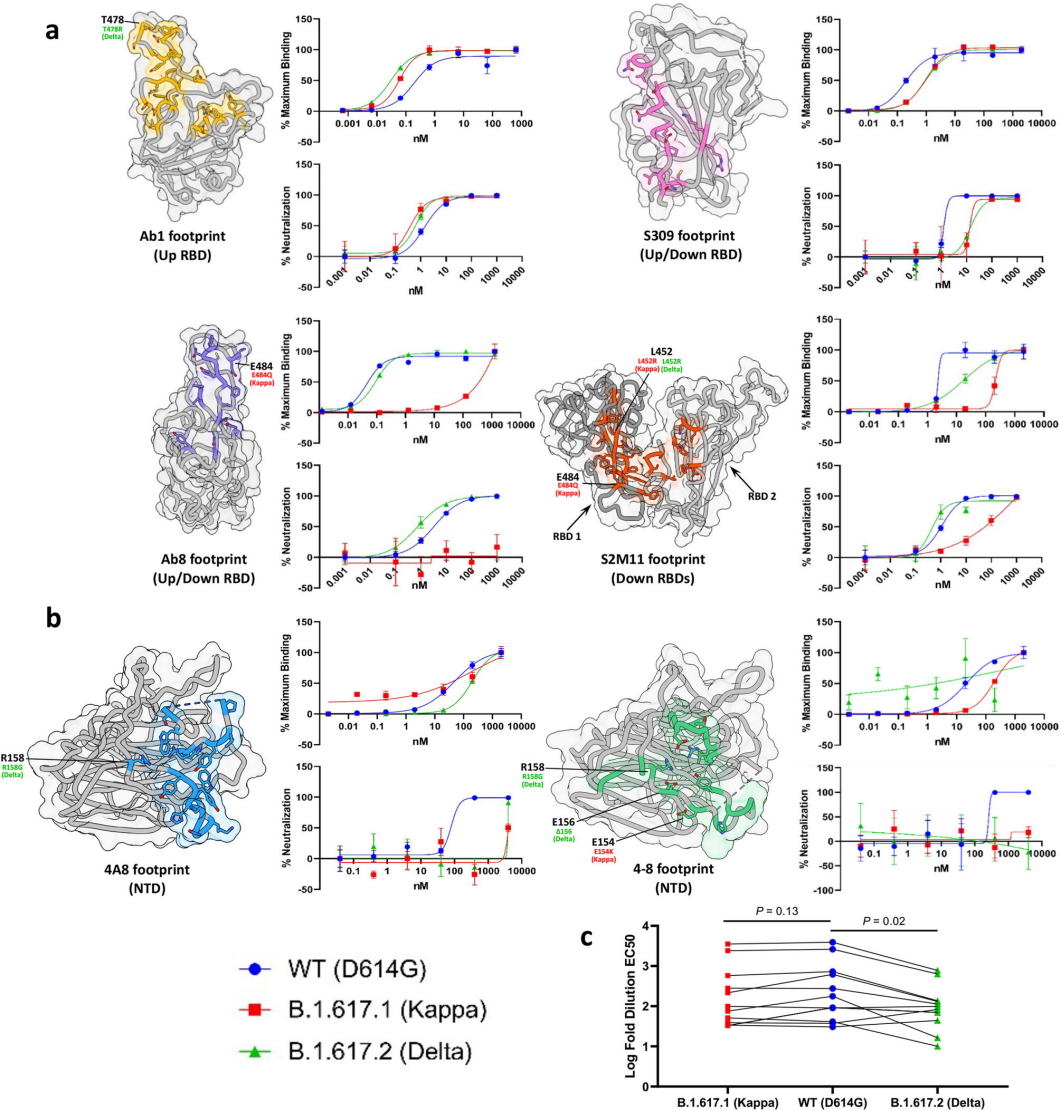
Antibody evasion by Delta and Kappa variants. **a** Antibody-binding footprints, antibody binding as assessed by ELISA, and pseudovirus neutralisation for four anti-RBD antibodies (Ab1 - PDB ID: 7MJJ, Ab8 - PDB ID: 7MJH, S309 – PDB ID: 6WPS, and S2M11 – PDB ID: 7K43). Both ELISA and pseudovirus neutralisation assays were performed at least in technical duplicate and the mean is plotted. Error bars represent standard deviation. **b** As in **a** but for two anti-NTD antibodies (4A8 - PDB ID: 7C2L and 4–8 - PDB ID: 7LQV) Both ELISA and pseudovirus neutralisation assays were performed at least in technical duplicate and the mean is plotted. Error bars represent standard deviation. **c** Log fold dilutions of sera conferring 50% neutralisation (EC_50_) of pseudovirus harbouring wild-type, Kappa or Delta spikes, from patients post COVID19 and/or vaccination (*n* = 12 patients). Statistical significance was tested via the Wilcoxon matched-pairs test (two-tailed). Patient-derived sera sample information and raw pseudovirus neutralisation data are presented in Supplementary Fig. 15. Source data are provided as a Source Data file.

To assess the antibody evasive properties of the Kappa and Delta spike protein mutations, we performed antibody binding and neutralisation studies using purified variant spike ectodomains (Supplementary Fig. 1) and variant pseudo-typed viruses respectively. First, a panel of six previously characterised monoclonal antibodies targeting epitopes in the spike RBD or NTD were assessed (Fig. 2a)^17–22^. Interestingly, we observed enhanced potency of ab1 for both Kappa and Delta variant spikes relative to wild-type, despite the presence of the Kappa T478K mutation which falls within the ab1 footprint. Analysis of the RBD-ab1 interface reveals close proximity of the ab1 amino-terminal glutamic acid to position 478 within the RBD (Supplementary Fig. 2). Thus, the enhanced potency of ab1 for the Delta variant is likely explained by the formation of an additional electrostatic interaction and/or salt bridge. We have previously demonstrated the sensitivity of ab8 and S2M11 to the E484K mutation, wherein both antibodies were fully escaped^23^. While total escape of ab8 was achieved by the E484Q-bearing Kappa variant spike, binding and neutralisation of S2M11 was attenuated but not abolished (Fig. 2a), demonstrating the relative sensitivities of these antibodies to mutation of E484. Although the S309 footprint does not include any Delta or Kappa RBD mutational positions, we observed reduced binding and neutralising potencies for both of these variant spikes relative to wild-type. The Kappa variant spike harbours mutations that fall within the footprints of both NTD-directed antibodies tested (4A8 and 4–8), and accordingly demonstrated total escape (Fig. 2b). The Delta spike contains NTD mutations within the footprint of 4–8 but not 4A8 and similarly evaded both antibodies (Fig. 2b), suggesting both direct and indirect mechanisms of evasion. Second, we evaluated the impact of Kappa and Delta spike protein mutations on neutralisation by polyclonal preparations of human antibodies. To this end, we utilised sera from a set of patients with varying vaccination statuses and COVID-19 histories (Supplementary Fig. 15) in pseudo-typed virus entry assays. We observed different degrees of attenuated neutralisation potencies for both variants when compared to wild-type and observed a statistically significant decrease in potency for the Delta variant (Fig. 2c, *p* = 0.02).

### Delta variant S protein mutations moderately increase ACE2 affinity as a consequence of enhanced electrostatic complementarity at the RBD-ACE2 interface

**Fig. 3:**
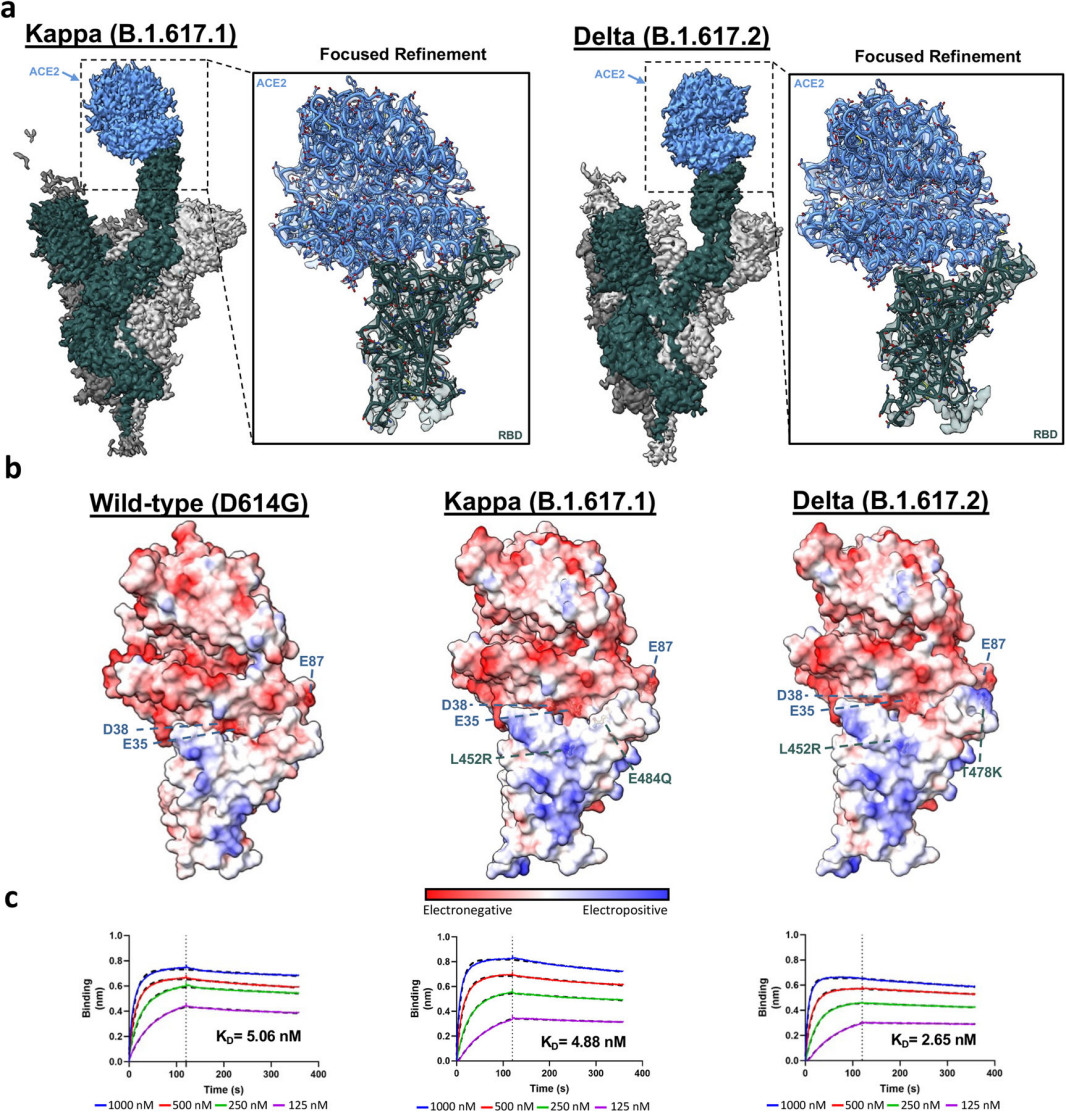
Structural and biophysical effects of Kappa and Delta S protein mutations on ACE2 binding. **a** The global and focus-refined cryo-EM structures for the Kappa and Delta variant S proteins in complex with ACE2. A more focused view to show the quality of the cryo-EM density at the S protein – ACE2 interface is provided in Supplementary Fig. 6. **b** Electrostatic surface potentials for the wild-type, Kappa and Delta S protein–ACE2 complexes. Electrostatic potential colouring spans −10 kcal mol^−1^ e^−1^ (darkest red) to 10 kcal mol^−1^ e^−1^ (darkest blue). We previously reported the structure of the wild-type S protein–ACE2 complex and we use this model here to generate the wild-type electrostatic surface^23^. **c** Biolayer interferometry (BLI) sensorgrams for the wild-type, Kappa, and Delta variant S protein–ACE2 binding interaction. The S protein concentrations utilised are: 1000 nM (blue), 500 nM (red), 250 nM (green), 125 nM (magenta). The *k*_on_ and k_off_ rate constants are presented in Supplementary Fig. 3. Source data are provided as a Source Data file.

As demonstrated in previously emerged SARS-CoV-2 variants of concern (Beta, Gamma), the evolution of the S protein seems to select for combinations of mutations that balance both antibody evasion and ACE2 binding affinity^23–25^. Having observed monoclonal and polyclonal antibody evasion for the Delta and Kappa variants (Fig. 2a, b), we next sought to assess the impact of these mutations on ACE2 affinity. Biolayer interferometry (BLI) analysis of trimeric variant S proteins revealed limited (no change) and moderate (~2-fold) enhancements in ACE2 affinity for the Kappa and Delta variants respectively (Fig. 3c and Supplementary Fig. 3). To investigate a structural rationale for the observed changes - or lack thereof - in ACE2 binding affinity, we solved the cryo-EM structures of the Kappa and Delta variant spike trimers in complex with ACE2 (Fig. 3a, Supplementary Figs. 4–6, and Supplementary Table 1).

The focus-refined atomic structure of the Kappa variant S protein in complex with ACE2 reveals limited structural changes at the RBD-ACE2 interface (Fig. 3a). The Kappa variant E484Q mutation results in the loss of an electrostatic interaction between residue E484 and residue K31 within ACE2, likely resulting in a weaker interaction at this site. However, the enhanced electrostatic complementarity afforded by the accompanying L452R mutation, as described previously, may present a compensatory mutation accounting for the lost E484–K31 interaction (Fig. 3b)^23^. The combination of these two opposing mutations, one diminishing ACE2 affinity (E484Q), and the other increasing ACE2 binding (L452R), is consistent with the unchanged overall affinity of the Kappa S protein–ACE2 binding interaction. Precedence for compensatory mutations towards increasing ACE2 affinity while decreasing antibody binding has been reported for the N501Y and K417N/T mutational combinations found in the Beta and Gamma variants^23–25^. Interestingly, the overall unchanged ACE2 affinity of the Kappa variant S protein stands in contrast to the majority of previously characterised SARS-CoV-2 variants of concern (Alpha, Beta, Gamma)^26–30^.

As for the Kappa variant, the Delta variant S protein–ACE2 complex focus-refined cryo-EM structure also reveals limited sidechain rearrangement at the RBD-ACE2 interface (Fig. 3b). The Delta variant lacks the E484Q substitution which preserves the E484–K31 electrostatic interaction, while the common L452R mutation may increase ACE2 binding by enhancing electrostatic complementarity^23^. Further, the Delta variant lysine substitution at position 478 (T478K) extends its positively charged sidechain towards an electronegative region on ACE2 (centred at position E87) (Fig. 3b). Therefore, the combination of enhanced electrostatic complementarity afforded by the L452R and T478K Delta variant substitutions likely accounts for the moderate increase in ACE2 affinity.

### Cryo-EM structure of the Kappa variant S protein reveals a head-to-head dimerisation phenotype

**Fig. 4:**
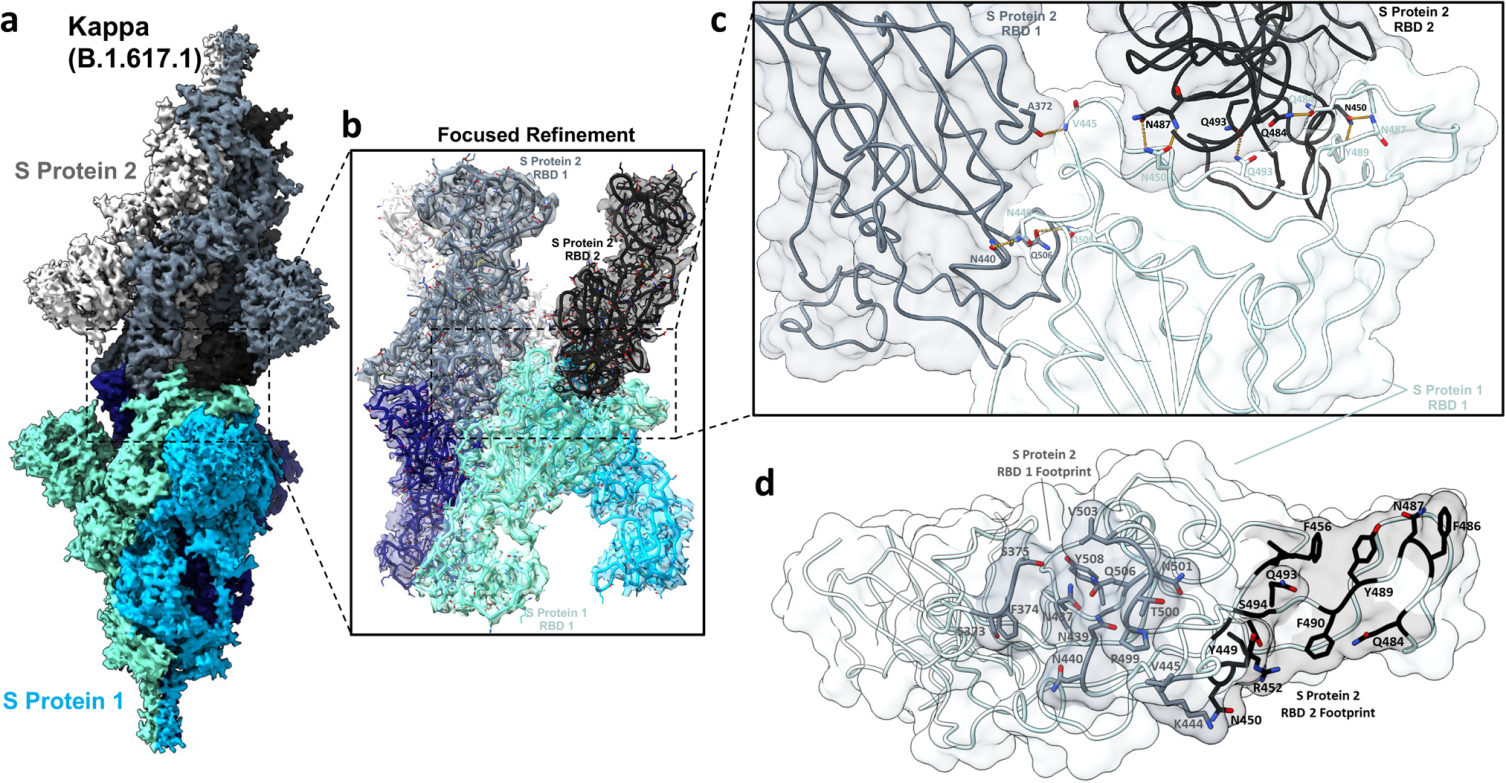
The Kappa variant S protein exhibits a novel dimer-of-trimers phenotype. **a** Side view of the global cryo-EM density map of the Kappa variant dimer-of-trimers complex. One trimer (S Protein 1, bottom) is displayed in various shades of blue, and the other trimer in grayscale (S Protein 2, top). **b** Focus-refined cryo-EM density map and fitted atomic model at the dimerisation interface. **c** Detailed view of the molecular interface between two RBDs in the grayscale trimer (top) and a single RBD in the blue shaded trimer (bottom). For amino acids involved in inter-residue hydrogen bonding, the backbone and sidechain atoms are displayed. Hydrogen bonds are indicated by yellow dashed lines. **d** The top-down view of the region in panel **c** is shown. The binding footprints of RBD1 and RBD2 are indicated by complementary shading and displaying the sidechain atoms of footprint residues.

We next aimed to evaluate the effect of Kappa and Delta S protein mutations on spike conformation and quaternary structure. We solved cryo-EM structures of the Kappa and Delta spikes at global average resolutions of 3.16 and 2.25 Å respectively (Supplementary Figs. 7 and 8 and Supplementary Table 1). The cryo-EM 3D reconstruction of the Delta spike reveals no large-scale structural changes, with the predominant conformation having one RBD in the up position, as found in the wild-type (D614G) construct (Supplementary Fig. 12). In contrast, the global reconstruction of the Kappa variant S protein uncovers a previously unreported head-to-head dimer-of-trimers phenotype (Fig. 4a). This dimer is mediated by RBD-RBD contacts between two S protein trimers, with one trimer offset at a 12° angle. This angle is a result of slightly asymmetric binding at each RBD-RBD contact. Focused refinement of the dimer-of-trimers interaction interface reveals an extensive binding interface involving all six RBDs (Fig. 4b). Each RBD interacts with two RBDs in the opposite trimer via two distinct interfaces, henceforth referred to as RBD1 and RBD2 (Fig. 4c, d). Interactions stabilising the RBD1 interface are primarily mediated by van der Waals interactions and hydrophobic contacts between residues across this interface. Additionally, homo- asparagine-asparagine and glutamine-glutamine hydrogen bonds at positions 440 and 506, respectively, and a backbone carbonyl oxygen–amide hydrogen bond between residues 372 and 445 further contribute to the RBD1 interface (Fig. 4c).

The second RBD-RBD interface (RBD2), comprises multiple doubly-hydrogen bonded residues, between residues N487-N450 and between residues N450-Y489/N487 (Fig. 4c, d). Additionally, two homo- glutamine-glutamine hydrogen bonds are present between residues Q493-Q493 and Q484-Q484 from each trimer. This latter interaction at position 484 is of particular interest as it is uniquely mutated from glutamic acid to glutamine (E484Q) in the Kappa variant. Given this unique substitution and the unique dimer-of-trimers phenotype seen only for the Kappa variant spike (and not for Alpha, Beta, Gamma, Epsilon and various RBD-mutated spike trimers), we identified position 484 as likely being crucial for S protein dimerisation^23^.

### Reduced charge repulsion and additional sidechain contacts at position 484 result in a dimer-of-trimers phenotype

**Fig. 5:**
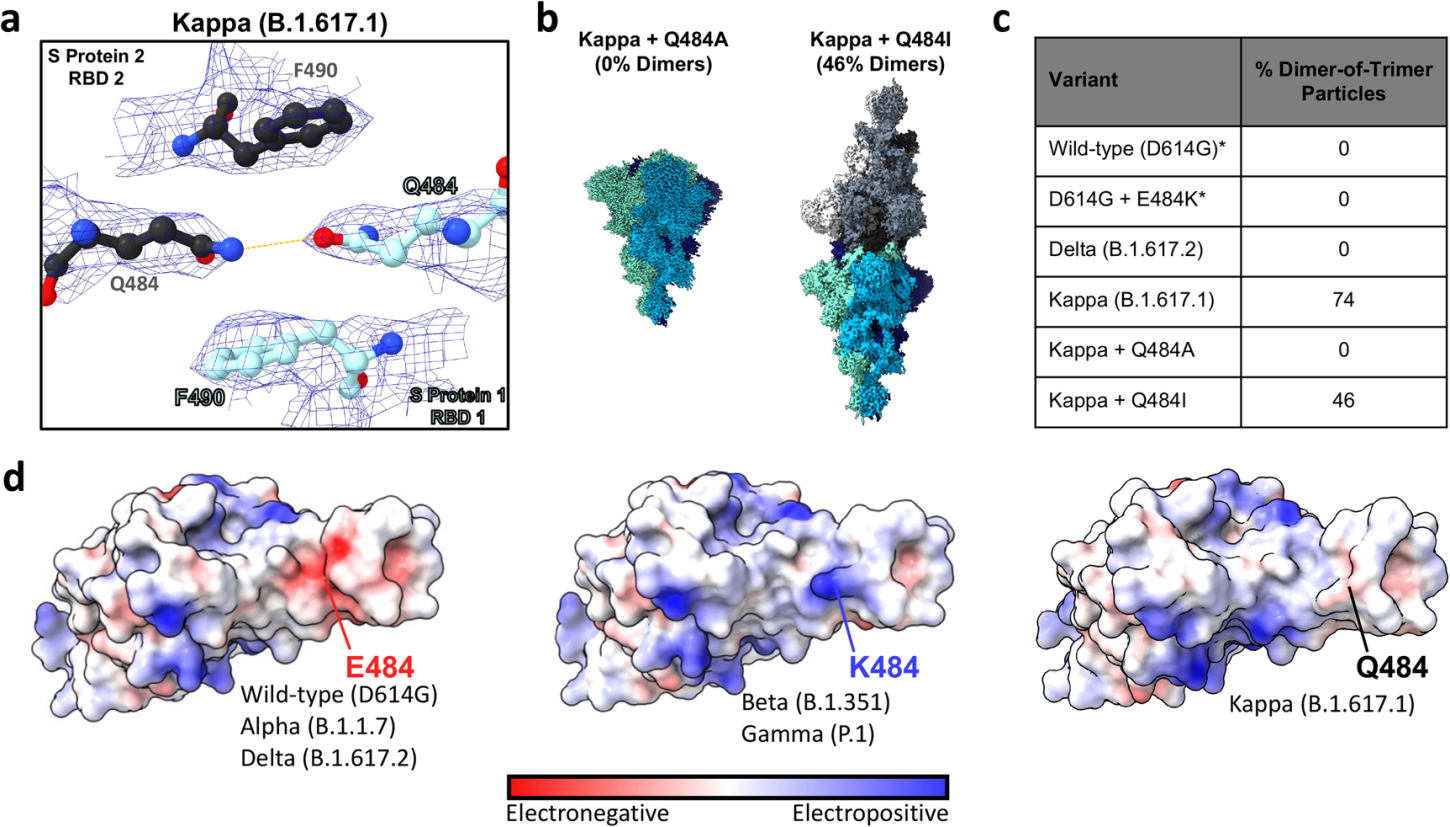
Impact of residue identity at position 484 on S protein oligomerization. **a** Detailed view of the Kappa (B.1.617.1) dimer-of-trimers cryo-EM density map and fitted model at the Q484-Q484 interaction site. The hydrogen bond formed between Q484 residues located in different S ectodomain trimers (S protein 2 RBD 2 in black, and S protein 1 RBD 1 in cyan) is indicated with a yellow dashed line. **b** Cryo-EM density maps for the Kappa + Q484A and Kappa + Q484I mutated S proteins. **c** Summary table of the proportion of dimerised particles in S proteins harbouring mutations at position 484. Asterisks indicate structures reported in previous publications^23^. **d** E484, K484, and Q484 RBD electrostatic surface potentials highlighting the surface potentials at position 484. The E484 surface potential was generated using the previously reported wild-type (D614G) + ACE2 focus-refined atomic model^23^. The K484 surface potential was generated using the previously reported D614G + N501Y + E484K focus-refined atomic model^23^. The Q484 surface potential was generated using the Kappa + ACE2 focus-refined atomic model reported in the present manuscript.

Having identified that residue identity at position 484 likely affects head-to-head S protein oligomerization, we aimed to further probe the chemical properties at 484 that mediate this dimerisation. A focused view of the Q484-Q484 hydrogen bond (Fig. 5a) shows the bond to be sandwiched by proximal bulky F490 aromatic sidechains. We therefore hypothesised that charge neutrality at position 484 (as seen in the Q484, but not E484 or the recently emerged K484 S proteins) may be sufficient to reduce charge–charge repulsion at this site and therefore allow dimerisation. To test this, we performed site-directed mutagenesis to substitute an alanine at position 484 (Q484A) in the Kappa S protein, purified the trimer, and performed structural studies. The cryo-EM reconstruction of Q484A spikes revealed no evidence of dimer-of-trimer assemblies, consistent with our previous results for wild-type and other variant of concern (VoC) spikes^23^ (Fig. 5b and Supplementary Fig. 9). We next hypothesised that the homo-glutamine hydrogen bond conferred by the Q484 sidechain provided an additional contact critical for dimer formation. Accordingly, we introduced the amino acid isoleucine at position 484, which possesses a branched aliphatic sidechain capable of providing hydrophobic packing contacts. The cryo-EM reconstruction yielded a dimer-of-trimers phenotype for the Kappa + Q484I S protein variant, yet, with a reduced number of picked particles comprising the dimer class (46%), relative to the original Kappa variant with Q484 (74%) (Fig. 5b, c and Supplementary Fig. 10). The Kappa and Kappa + Q484I dimer-of-trimers are structurally very similar, with RMSD values of 0.262 Å and 0.705 Å for the global and focus-refined atomic models, respectively (Supplementary Fig. 13). The oligomerization state of S proteins harbouring charged residues at 484 (E484, K484), along with the Q484A and Q484I mutations demonstrate that abrogation of charge at position 484 is necessary but not sufficient to permit dimerisation. Rather, a combination of charge neutralisation and additional contacts enabled by sidechains at position 484 is required for S protein dimerisation.

An analysis of the electrostatic surface potential at position 484 of the RBD reveals a unique property of the Kappa variant that may explain its propensity to dimerise. Figure 5d shows that the wild-type/Alpha/Delta variants and the Beta/Gamma variants may be binned into electronegative and electropositive surfaces at position 484, respectively, which would result in charge–charge repulsion if these variant S proteins were to dimerise in the same manner as the Kappa variant. The Kappa variant uniquely has an absence of charge at position 484 in its S protein, as reflected in the neutral surface potential shown in Fig. 5d, consistent with its distinguishing ability to form head-to-head dimers.

## Discussion

Herein, we present a comparative analysis of the effects of the Delta and Kappa variant S protein mutations on aspects of viral fitness. Our overarching finding, as discussed below, is that in the context of antibody evasion and ACE2 affinity, the Delta and Kappa variants do not differ to a great degree, and that the global dominance of the Delta variant is likely a result of enhancements in other aspects of viral fitness. Here, we discuss findings reported by others examining different aspects of viral fitness to rationalize the success of the Delta variant. Additionally, we highlight the Kappa variant S protein dimerisation as a distinguishing feature unique to this variant spike.

Antibody neutralisation escape is an important element of increased viral fitness, with all previously characterised variants of concern exhibiting some degree of antibody escape^26,27,30–32^. Our antibody binding and neutralisation experiments demonstrate that both variants escape monoclonal antibodies (via direct or potentially allosteric mutational mechanisms) and that these variants evade neutralisation by vaccine-induced polyclonal antibody sera. These results are broadly consistent with initial reports on Kappa and Delta variant antibody escape^9,16,33,34^. Interestingly, a recent preprint - which includes a large panel of vaccine-induced sera samples - reports greater antibody evasion for the Kappa variant spike over the Delta variant^10^. There are several similar or identical mutations common between the Kappa/Delta variants and previously emerged variants of concern (Alpha, Beta, Gamma and Epsilon). For example, the L452R mutation, mutations at position 484 (E484K and E484Q) and mutations within the N3 loop in the NTD (residues 141–156) have all been previously characterised for their antibody-evasive effects in other variants. Our results demonstrating conserved mutational effects on antibody escape for these mutations in the Kappa and Delta variants – as seen in previously characterised variants of concern – further substantiates the modular nature of these mutations^23^.

We found unchanged and moderately increased ACE2 affinities for the Kappa and Delta variant spikes (compared to wild-type S protein) respectively. However, this moderate (~2-fold) increase in ACE2 binding does not rationalize the dominance of the Delta variant over other variants with higher fold-changes in ACE2 affinities (Alpha, Beta, Gamma)^26–30^. Analysis of the electrostatic surface potential of the ACE2-bound Delta variant S protein is consistent with enhancements in electrostatic complementarity afforded by both the L452R and T478K mutations. The same L452R mutation in the Kappa variant likely did not result in an overall change in ACE2 affinity due to the deleterious effects of a lost electrostatic interaction from the E484Q mutation. The only other study reporting Kappa/Delta S protein–ACE2 affinity differences (using RBDs alone) found effectively no change in affinity across enzyme-linked immunosorbent assay (ELISA), SPR and BLI analyses^10^. Mutations outside of the RBD have however been demonstrated to influence ACE2 engagement by modulating RBD conformation, such as the D614G and A570D mutations in the B.1 and B.1.1.7 (Alpha variant) lineages respectively^4,25,35,36^. Therefore, the use of trimeric S proteins as employed in the present study may be important to realise allosteric effects on ACE2 binding by Kappa and Delta mutations outside of the RBD.

Biochemical analyses of the Kappa and Delta variant S proteins provide insights into the behaviour of the novel Delta plus (B.1.617.2+) variant. The Delta plus variant contains the same mutations as the Delta variant, with the addition of a lysine to asparagine substitution at position 417 (K417N). The K417N mutation is found identically (K417N) and similarly (K417T) in the Beta (B.1.351) and Gamma (P.1) VoCs respectively^1–3^. This mutation in isolation has previously been shown to enhance antibody evasion and concomitantly decrease ACE2 binding affinity^23^. The deleterious effect of decreased ACE2 affinity imparted by K417N/T mutation is compensated by N501Y substitution in Beta and Gamma VoCs, but not in Delta plus, potentially rationalising its relatively limited global prevalence^37,38^. Consistent with previous mutational studies that used S proteins with the K417N mutation alone, recent results have demonstrated enhanced immune evasion and decreased ACE2 affinity for the Delta plus (B.1.617.2+) variant relative to Delta (B.1.617.2)^10^. The consistency of the effects imparted by the K417N substitution, in isolation or when combined with other mutations, consolidates the understanding of naturally selected mutations as mostly independent functional modules at the molecular level. On this basis, the effects of rapidly emerging mutations that occur at a known location in the protein sequence can be accurately inferred from the in-depth structural and biophysical characterisation of previous variants – even when these VoCs do not belong to the same lineage. Therefore, continued characterisation of the effects of individual and combinations of mutations on overall viral fitness will allow researchers to better and more rapidly predict the behaviour of future emerging SARS-CoV-2 variants – with potential implications for vaccine modification and booster regimes.

The antibody evasion and ACE2 binding effects imparted by the Delta variant mutations are not unique to this VoC, yet the Delta variant has largely eclipsed other VoCs in terms of global prevalence (Fig. 1d). Therefore, it is likely that mutational effects that influence other aspects of viral fitness (not assessed in the present publication) further rationalize the unique current prevalence of the Delta lineage. For example, the P681R mutation - found in all B.1.617 sub-lineages - has recently been reported to enhance S protein cleavage and cell-cell fusion^11,15,34^. As S protein cleavage is a crucial step in the pre- to post-fusion transition of the S protein, the P681R mutation likely contributes to the reported increase in Delta variant replication kinetics and viral loads in oropharyngeal and nose/throat swabs of infected individuals^16,39^. However, given the shared P681R mutation in both Kappa and Delta variants, this mutation alone does not explain the prevalence of the Delta variant over Kappa in India initially and subsequently globally. Finally, the effects of the D950N mutation (Delta) and the Q1071H mutation within the S protein fusion machinery (Kappa), as well as mutations outside of the S protein open-reading frame, have yet to be assessed for their impact on SARS-CoV-2 viral fitness.

Consistent with published structures of other SARS-CoV-2 VoC S proteins, our structure of the Delta S protein revealed no large changes in global 3D structure (Supplementary Fig. 12). However, for the Kappa variant we report an unprecedented head-to-head S trimer dimerisation, likely facilitated by the abrogation of charge–charge repulsion and the additional contacts afforded by the E484Q substitution. Our synthetic mutation of Q484I in the Kappa variant background, which also resulted in dimerisation, suggests that the SARS-CoV-2 S protein may be a single amino acid substitution away from exhibiting this dimer-of-trimers phenotype. However, an analysis of mutational frequency at position 484 in globally deposited sequences reveals that only E484, K484 and Q484 S protein genotypes have ever been present at >1% of total deposited sequences, suggesting limited mutational flexibility at this position (Supplementary Fig. 14). We found this head-to-head dimerisation to be concentration-dependent, with no evidence of dimerisation in experiments conducted at <0.05 mg/mL (see size-exclusion chromatography, Supplementary Fig. 1, and negative-stain electron microscopy, Supplementary Fig. 11). Thus, we hypothesise that if local spike concentrations reach high enough concentrations to dimerise at any point during the SARS-CoV-2 cell entry, replication and packaging events, this dimerisation phenomenon could have biological implications. When considering membrane-embedded S protein dimerisation, cryo-electron tomography results from Ke et al.^40^ and Yao et al.^41^ suggest that the S protein adopts angles relative to the viral membrane that would be incompatible with intra-virion S protein dimerisation, therefore if dimerisation were to take place in physiological and membrane-embedded contexts, it would likely be limited to between viral particles (inter-virion)^40,41^.

While future studies will be required to assess the potential biological relevance – if any – of the reported dimerisation of spike trimers, we speculate here on some mechanisms by which dimerised S proteins could theoretically result in increased or decreased viral fitness. Firstly, head-to-head S protein dimerisation buries much of the antibody-accessible surface area of the RBD (the predominant target of neutralising antibodies) and could shield this otherwise vulnerable neutralisation site^13^. Secondly, dimerised spikes - in the same manner as reported for the Kappa variant - would be unable to engage the ACE2 receptor and therefore not be able to enter host cells through the ACE2-dependent cell-entry pathway^42^. These first two competing mechanisms resulting in increased and decreased viral fitness, respectively, could favour a spike protein with a finely tuned balance of dimerisation potential to both mask neutralising epitopes, but also to readily dissociate and permit engagement of ACE2. To this second point, we verified that the Kappa S protein dimer-of-trimers complex is labile enough to still permit ACE2 binding through our experimental derivation of the ACE2 bound structure. We saw no evidence of S protein dimer-of-trimer formation in our cryo-EM images upon introducing a modest excess of ACE2 (~1:1.25 S protein trimer : ACE2 molar ratio), despite an identical S protein concentration which resulted in the dimer-of-trimers reconstruction. Interestingly, two recent publications have independently described potent neutralising nanobodies with propensities to induce S protein dimers^43,44^. While the exact mode of neutralisation for these nanobodies remains unclear, this may suggest that S protein dimerisation has negative impacts on viral fitness. Further studies to elucidate the biological implications, if any, of this dimerisation phenomenon are therefore highly relevant in the rapidly evolving SARS-CoV-2 variant landscape.

### Limitations

In the present study, we have assessed the impact of Delta and Kappa variant mutations on S protein structure, ACE2 binding and antibody evasion – three important but not comprehensive aspects of S protein fitness. Additionally, we have not assessed Delta and Kappa mutations outside of the S protein open-reading frame for their potential impacts on viral fitness. Our analyses make use of HexaPro constructs, which differ from native S proteins by the incorporation of six stabilising proline substitutions (F817P, A892P, A899P, A942P, K968P and V969P) and the replacement of the transmembrane domain with a trimerization motif^45^. These protein modifications result in substantially higher yields during mammalian expression and increased thermostability, which facilitate the biochemical experiments presented here. With regards to the Kappa variant head-to-head dimer-of-trimers phenomenon, we acknowledge that this interaction may only occur under the highly concentrated and vitrified cryo-EM sample conditions employed in this study, which may not represent natural biological contexts. Future studies will be required to assess any potential impacts of S protein dimerisation on viral pathobiology.

## Methods

### Biological materials availability

All unique biological materials described in this manuscript are made available upon reasonable request from the corresponding author and following the completion of a materials transfer agreement.

### Pseudovirus neutralization assay

SARS-CoV-2 S protein Delta and Kappa genes were synthesised and inserted into pcDNA3.1 (GeneArt Gene Synthesis, Thermo Fisher Scientific). The production of the SARS-CoV-2 wild-type (D614G) S protein was described previously^23^. Variant pseudotyped retroviral particles were produced in HEK293T cells as described previously^46^. Briefly, a third-generation lentiviral packaging system was utilised in combination with plasmids encoding the full-length SARS-CoV-2 spike, along with a transfer plasmid encoding luciferase and GFP as a dual reporter gene. Pseudoviruses were harvested 60 h after transfection, filtered with a 0.45 µm PES filter, and frozen. For neutralization assays, HEK293T-ACE2-TMPRSS2 cells^47^ (BEI Resources cat# NR-55293) were seeded in 96- or 384-well plates at 50,000 or 20,000 cells respectively. The next day, pseudovirus preparations normalised for viral capsid p24 levels (Lenti-X™ GoStix™ Plus) were incubated with dilutions of the indicated antibodies or sera (obtained under the approval of the UBC Clinical Research Ethics Board), or media alone for 1 h at 37 °C prior to addition to cells and incubation for 48 h. Cells were then lysed and luciferase activity assessed using the ONE-Glo™ EX Luciferase Assay System (Promega) according to the manufacturer’s specifications. Detection of relative luciferase units was carried out using a Varioskan Lux plate reader (Thermo Fisher). Percent neutralisation was calculated relative to signals obtained in the presence of virus alone for each experiment. The kits, reagents and DNA described in this section are summarised in Supplementary Tables 2 and 6.

### Expression and purification of recombinant spike protein constructs

The wild-type SARS-CoV-2 S HexaPro expression plasmid was previously described^45^ and was a gift from Jason McLellan (Addgene plasmid #154754; http://n2t.net/addgene:154754; RRID:Addgene_154754).

The VoC RBD mutations were introduced by site-directed mutagenesis (Q5 Site-Directed Mutagenesis Kit, New England Biolabs). Successful cloning was confirmed by Sanger sequencing (Genewiz, Inc.). The following primers were used (5′–3′):

B.1.617.1 E484A HexaPro

Forward: TAATGGCGTGgcgGGCTTCAACTGCTACTTCCCAC

Reverse: CAAGGGGTGCTGCCGGCC

B.1.617.1 E484I HexaPro

Forward: TAATGGCGTGatcGGCTTCAACTGCTACTTCCCACTG

Reverse: CAAGGGGTGCTGCCGGCC

Expi293F cells (Thermo Fisher, Cat# A14527, Supplementary Table 5) were grown in suspension culture using Expi293 Expression Medium (Thermo Fisher, Cat# A1435102) at 37 °C, 8% CO_2_. Cells were transiently transfected at a density of 3 × 10^6^ cells/mL using linear polyethylenimine (Polysciences Cat# 23966-1). The media was supplemented 24 h after transfection with 2.2 mM valproic acid, and expression was carried out for 3–5 days at 37 °C, 8% CO_2_. The supernatant was harvested by centrifugation and filtered through a 0.22-μM filter prior to loading onto a 5 mL HisTrap excel column (Cytiva). The column was washed for 20 CVs with wash buffer (20 mM Tris pH 8.0, 500 mM NaCl), 5 CVs of wash buffer supplemented with 20 mM imidazole and the protein eluted with elution buffer (20 mM Tris pH 8.0, 500 mM NaCl, 500 mM imidazole). Elution fractions containing the protein were pooled and concentrated (Amicon Ultra 100 kDa cut off, Millipore Sigma) for gel filtration. Gel filtration was conducted using a Superose 6 10/300 GL column (Cytiva) pre-equilibrated with GF buffer (20 mM Tris pH 8.0, 150 mM NaCl). Peak fractions corresponding to soluble protein were pooled and concentrated to 4.5–5.5 mg/mL (Amicon Ultra 100 kDa cut off, Millipore Sigma). Protein samples were flash-frozen in liquid nitrogen and stored at −80 °C.

### Antibody production

VH-FC ab8, IgG ab1, Fab S309, and Fab S2M11 were produced as previously described^17,18^. Plasmids encoding the light and heavy chains for Fab 4A8 and Fab 4–8 were synthesised (GeneArt Gene Synthesis, Thermo Fischer Scientific). Heavy chains were designed to incorporate a C terminal 6x histidine tag. Expi293 cells were transfected at a density of 3 × 10^6^ cells/mL using linear polyethylenimine (Polysciences Cat# 23966-1). In all, 24-h following transfection, media was supplemented with 2.2 mM valproic acid, and expression was carried out for 3–5 days at 37 °C, 8% CO_2_. The supernatant was harvested by centrifugation and filtered through a 0.22-μM filter prior to loading onto a 5 mL HisTrap excel column (Cytiva). The column was washed for 20 CVs with wash buffer (20 mM Tris pH 8.0, 500 mM NaCl), 5 CVs of wash buffer supplemented with 20 mM imidazole. The protein was eluted with elution buffer (20 mM Tris pH 8.0, 500 mM NaCl, 500 mM imidazole). Elution fractions containing the protein were pooled and concentrated (Amicon Ultra 10 kDa cut off, Millipore Sigma) for gel filtration. Gel filtration was conducted using a Superose 6 10/300 GL column (Cytiva) pre-equilibrated with GF buffer (20 mM Tris pH 8.0, 150 mM NaCl). Peak fractions corresponding to soluble protein were pooled and concentrated to 8–20 mg/mL (Amicon Ultra 10 kDa cut off, Millipore Sigma). Protein samples were stored at 4 °C until use. The antibodies described in this section are summarised in Supplementary Table 3.

### Electron microscopy sample preparation and data collection

For cryo-EM, S protein samples were prepared at 2.25 mg/mL, with and without the addition of ACE2 (Supplementary Table 4) at 0.5 mg/mL (1:1.25S protein trimer:ACE2 molar ratio). Vitrified samples of all S protein samples were prepared by first glow discharging Quantifoil R1.2/1.3 Cu mesh 200 holey carbon grids for 30 s using a Pelco easiGlow glow discharge unit (Ted Pella) and then applying 1.8 µL of protein suspension to the surface of the grid at a temperature of 10 °C and a humidity level of >98%. Grids were blotted (12 s, blot force −10) and plunge frozen into liquid ethane using a Vitrobot Mark IV (Thermo Fisher Scientific). All cryo-EM samples were imaged using a 300 kV Titan Krios G4 transmission electron microscope (Thermo Fisher Scientific) equipped with a Falcon4 direct electron detector in electron event registration (EER) mode. Movies were collected at ×155,000 magnification (calibrated pixel size of 0.5 Å per physical pixel) over a defocus range of −0.5 µm to −3 µm with a total dose of 40 e^−^/Å^2^ using EPU automated acquisition software. For negative stain, S protein samples were prepared at 0.1 mg/mL. Grids of Cu mesh 300 with continuous ultra-thin carbon film (CF300-Cu-UL, Electron Microscopy Sciences) were glow discharged for 15 s using the Pelco easiGlow. Samples were allowed to adsorb for 30 s before blotting away excess liquid, followed by a brief wash with MilliQ H_2_O. Grids were stained by three successive applications of 2% (w/v) uranyl formate (20 s, 20 s, 60 s). Negative stain grids were imaged using a 200 kV Glacios transmission electron microscope (Thermo Fisher Scientific) equipped with a Falcon3 camera operated in linear mode. Micrographs were collected using EPU at ×92,000 magnification (physical pixel size 1.6 Å) over a defocus range of −1 µm to −2 µm with a total accumulated dose of 120 e^−^/Å^2^. The software and algorithms described in this section are summarised in Supplementary Table 7.

### Image processing

The detailed workflow for the data processing is summarised in Supplementary Figs. 4–10. In general, all data processing was performed in cryoSPARC v.3.2^48^ unless stated otherwise. Motion correction in patch mode (EER upsampling factor 1, EER number of fractions 40), CTF estimation in patch mode, reference-free particle picking, and particle extraction were performed on-the-fly in cryoSPARC. After preprocessing, particles were subjected to 2D classification and/or 3D heterogeneous classification. The final 3D refinement included per particle CTF estimation and aberration correction. For B.1.617.1 spike proteins, focused refinements were performed with a soft mask covering all six RBDs. For the complexes of spike protein ectodomain and human ACE2, focused refinements were performed with a soft mask covering a single RBD and its bound ACE2. Global resolution and focused resolution were determined according to the gold-standard FSC^49^. The software and algorithms described in this section are summarised in Supplementary Table 7.

### Model building and refinement

For models of spike protein ectodomain alone, the SARS-CoV-2 HexaPro S trimer with N501Y mutation (PDB code 7MJG) was docked into the cryo-EM density map using UCSF Chimera v.1.15^50^. Then, mutation and manual adjustment were done with COOT v.0.9.3^51^, followed by iterative rounds of refinement in COOT and Phenix v.1.19^52^. Glycans were added at N-linked glycosylation sites in COOT. For models of complex of spike protein ectodomain and human ACE2, the RBD-ACE2 subcomplex was built using published coordinates (PDB code 7MJN) as the initial model, followed by refinement against focused refinement maps. The obtained model was then docked into global refinement maps together with the other individual domains of the spike protein. Model validation was performed using MolProbity^53^. Figures were prepared using UCSF Chimera, UCSF ChimeraX v.1.1.1^54^ and PyMOL (v.2.2 Schrodinger, LLC). The software and algorithms described in this section are summarised in Supplementary Table 7.

### Biolayer interferometry S protein-ACE2 binding assay

The kinetics of SARS-CoV-2 trimers and human ACE2 binding were analysed with the biolayer interferometer BLItz (ForteBio, Menlo Park, CA). Protein-A biosensors (ForteBio: 18–5010) were coated with ACE2-mFc (40 µg/mL) for 2 min and incubated in DPBS (pH = 7.4) to establish baselines. Concentrations of 125, 250, 500, and 1000 nM spike trimers were used for association for 2 min followed by dissociation in DPBS for 5 min. The association (*k*_on_) and dissociation (*k*_off_) rates were derived from the fitting of sensorgrams and used to calculate the binding equilibrium constant (K_D_).

### Enzyme-linked immunosorbent assay (ELISA)

For ELISA, 100 µL of wild-type (D614G), Kappa or Delta SARS-CoV-2 S proteins were coated onto 96-well MaxiSorp™ plates at 2 µg/mL in PBS overnight at 4 °C. All washing steps were performed three times with PBS + 0.05% Tween 20 (PBS-T). After washing, wells were incubated with blocking buffer (PBS-T + 1% casein) for 1 h at room temperature. After washing, wells were incubated with dilutions of primary antibodies in PBS-T + 0.5% BSA buffer for 1 h at room temperature. After washing, wells were incubated with goat anti-human IgG (Jackson ImmunoResearch) at a 1: 8000 dilution in PBS-T + 1% casein buffer for 1 h at room temperature. After washing, the substrate solution (Pierce™ 1-Step™) was used for colour development according to the manufacturer’s specifications. Optical density at 450 nm was read on a Varioskan Lux plate reader (Thermo Fisher Scientific).

### Reporting summary

Further information on research design is available in the Nature Research Reporting Summary linked to this article.

## Supplementary information

Supplementary Information

Reporting Summary

## Data Availability

The data that support this study are available from the corresponding author upon reasonable request. The atomic models and cryo-EM density maps have been deposited into the Protein Data Bank (PDB) and Electron Microscopy Data Bank (EMDB) as follows: Kappa spike dimer-of-trimers global refinement: PDB 7TF5 and EMD-25862, Kappa spike dimer-of-trimers focused refinement: PDB 7TF4 and EMD-25861, Kappa spike + ACE2 global refinement: PDB 7TF0 and EMD-25857, Kappa spike + ACE2 focused refinement: PDB 7TEZ and EMD-25856, Kappa spike + Q484A: PDB 7TF3 and EMD-25860, Kappa spike + Q484I global refinement: PDB 7TF2 and EMD-25859, Kappa spike + Q484I focused refinement: PDB 7TF1 and EMD-25858, Delta spike global refinement: PDB 7TEY and EMD-25855, Delta spike + ACE2 global refinement: PDB 7TEX and EMDB-25854, and Delta spike + ACE2 focused refinement: PDB 7TEW and EMD-25853. Source data are provided with this paper.

## Source data

Source Data
