# Supplementary material for: Structural and biochemical rationale for enhanced spike protein fitness in delta and kappa SARS-CoV-2 variants
Source: Nat Commun. 2022 Feb 8;13:742. doi: 10.1038/s41467-022-28324-6 (PMC8826856; doi:10.1038/s41467-022-28324-6)
Supplement: Supplementary file 1 — Supplementary Information [file 41467_2022_28324_MOESM1_ESM.pdf]

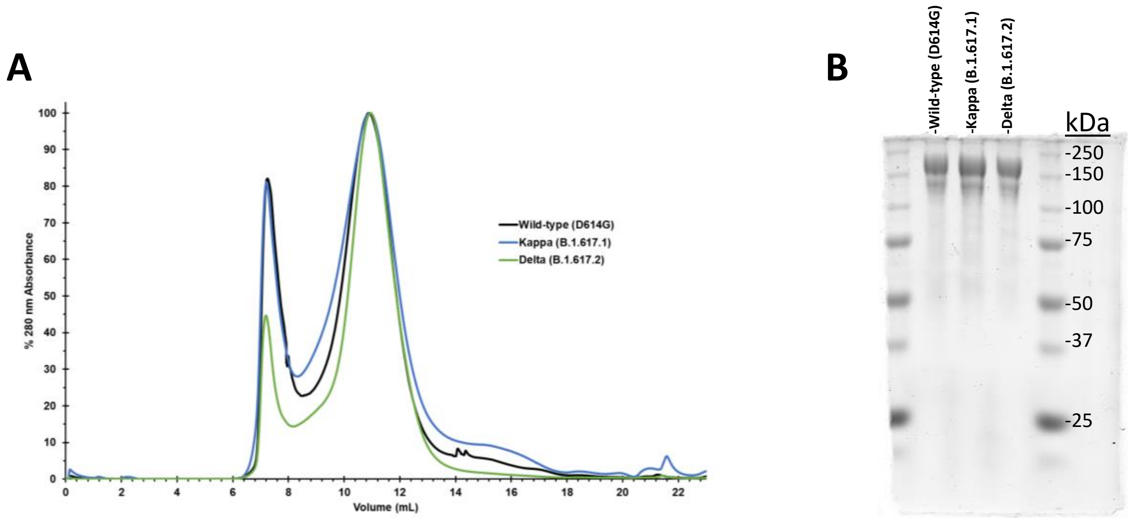

Supplementary Figure 1: Purification of wild-type (D614G), Kappa (B.1.617.1), and Delta (B.1.617.2) spike protein ectodomains. (A) Superose 6 10/300 GL size-exclusion traces for the spike protein ectodomains employed in this study. **(B)** SDS polyacrylamide gel electrophoresis (SDS-PAGE) gel of the three SARS-CoV-2 S proteins employed in this study. A normalized amount (4 µg) of each variant spike protein was loaded in each well. Proteins were analyzed via SDS-PAGE once. Source data are provided as a Source Data file.

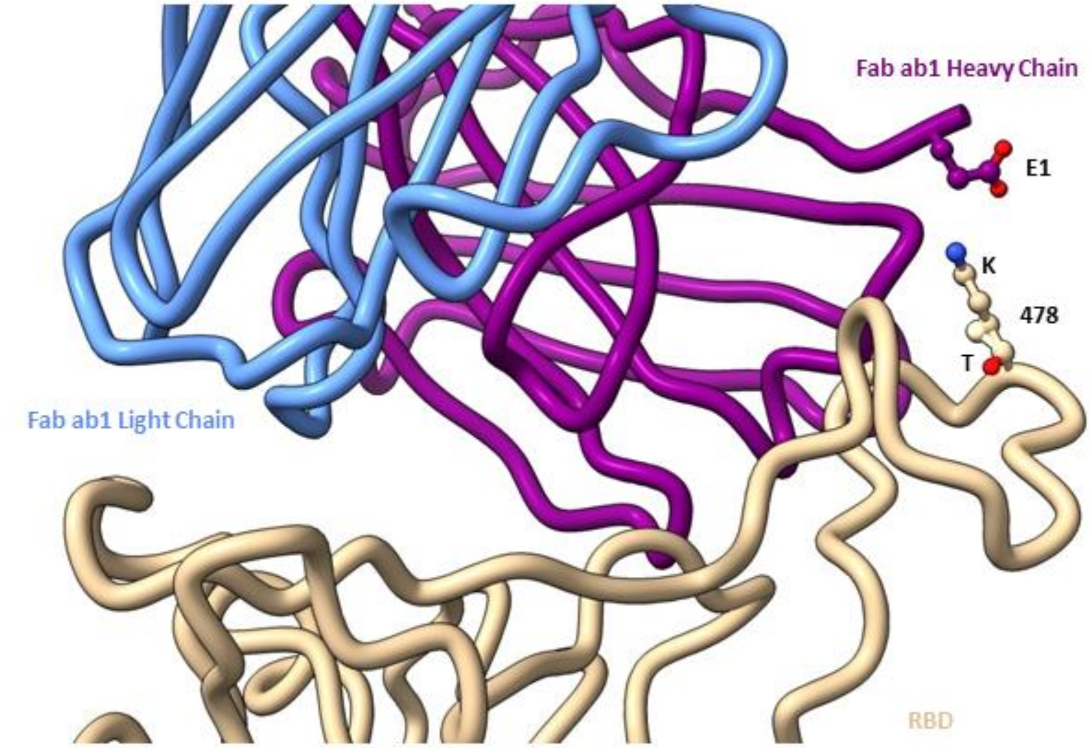

Supplementary Figure 2: Proximity of position 478 within the Delta (B.1.617.2) Variant RBD to Glutamic acid 1 within Fab ab1. The model resulting from focused refinement of the interface between the N501Y mutant spike and Fab ab1 was used (PDB: 7MJL). The proposed positioning of K478 is shown overlapping the experimental positioning of T478.

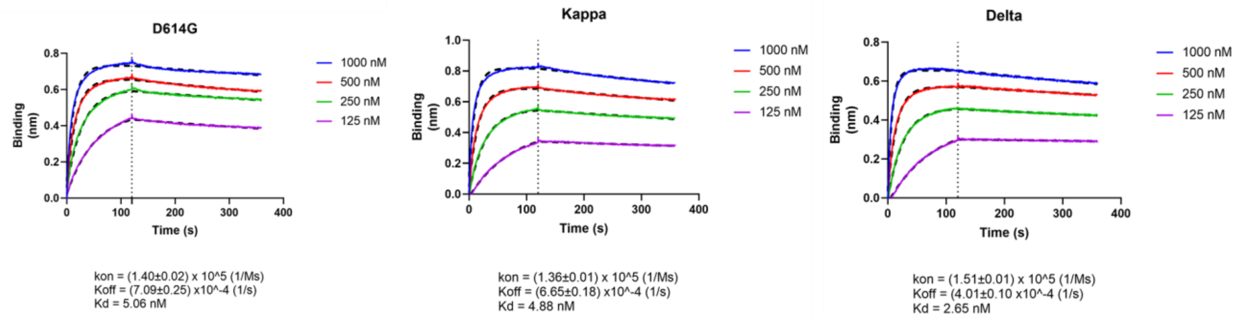

Supplementary Figure 3: Raw biolayer interferometry (BLI) sensorgrams for ACE2 binding to wild-type (D614G), Kappa (B.1.617.1), and Delta (B.1.617.2) spike protein ectodomains. ACE2 was immobilized on BLI sensor tips and S proteins were assessed for binding at different concentrations as indicated. Biophysical parameters (K_D, k_{on}, k_{off}) are shown as means. Errors correspond to standard deviations.

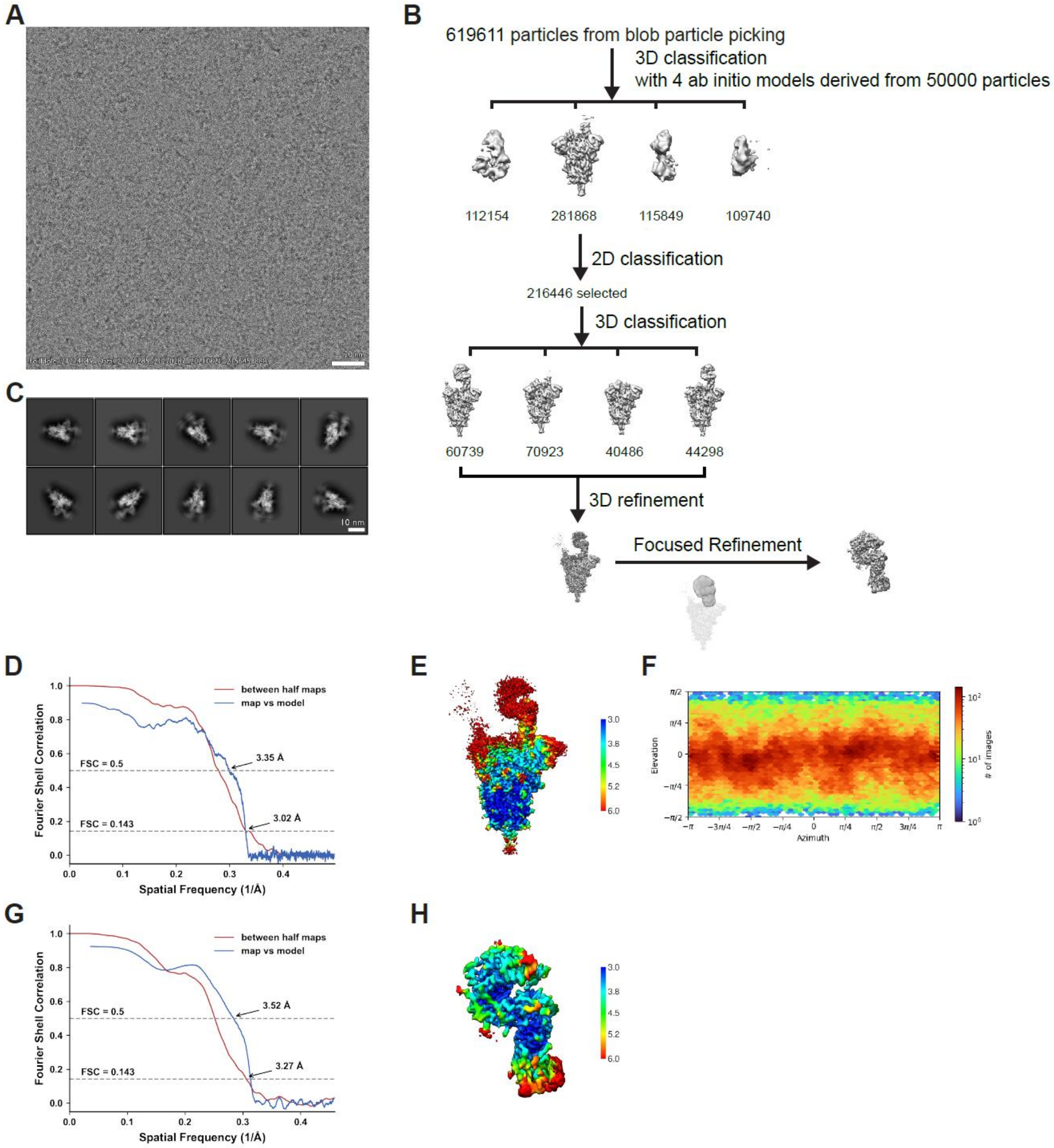

Supplementary Figure 4. Cryo-EM data processing and validation for the Kappa (B.1.617.1) spike protein ectodomain in complex with ACE2. (A) Representative cryo-EM micrograph from 8478 micrographs. **(B)** Workflow of cryo-EM image processing. **(C)** Representative 2D classes. **(D)** Fourier Shall Correlation (FSC) curves for the global refinement. **(E)** Local resolution map for the global refinement. **(F)** Viewing direction distribution plot. **(G)** Fourier Shall Correlation (FSC) curves for the focused refinement of the RBD-ACE2 interface. **(H)** Local resolution map for the focused refinement.

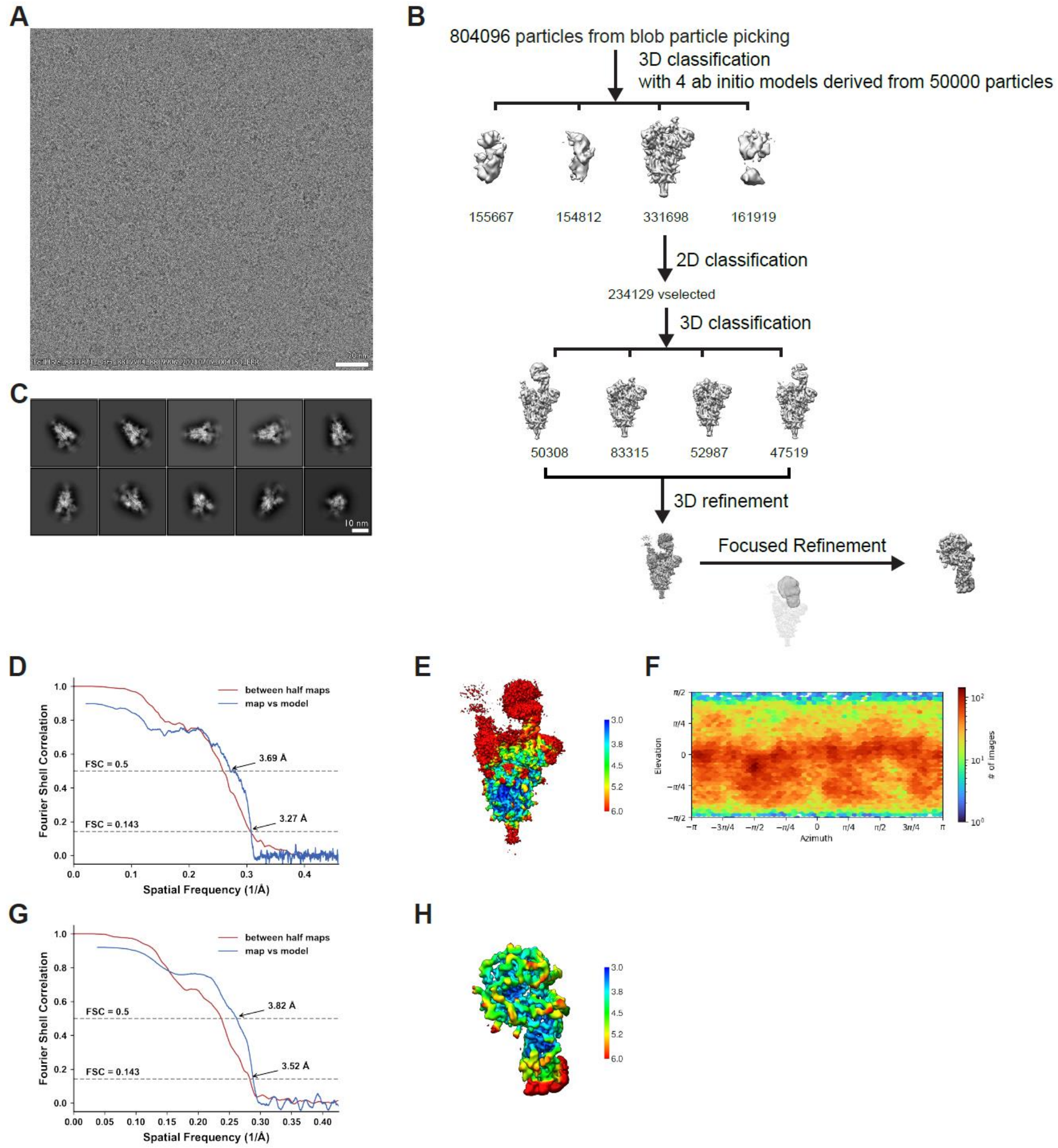

Supplementary Figure 5. Cryo-EM data processing and validation for the Delta (B.1.617.2) spike protein ectodomain in complex with ACE2. (A) Representative cryo-EM micrograph from 9630 micrographs. **(B)** Workflow of cryo-EM image processing. **(C)** Representative 2D classes. **(D)** Fourier Shall Correlation (FSC) curves for the global refinement. **(E)** Local resolution map for the global refinement. **(F)** Viewing direction distribution plot. **(G)** Fourier Shall Correlation (FSC) curves for the focused refinement of the RBD-ACE2 interface. **(H)** Local resolution map for the focused refinement.

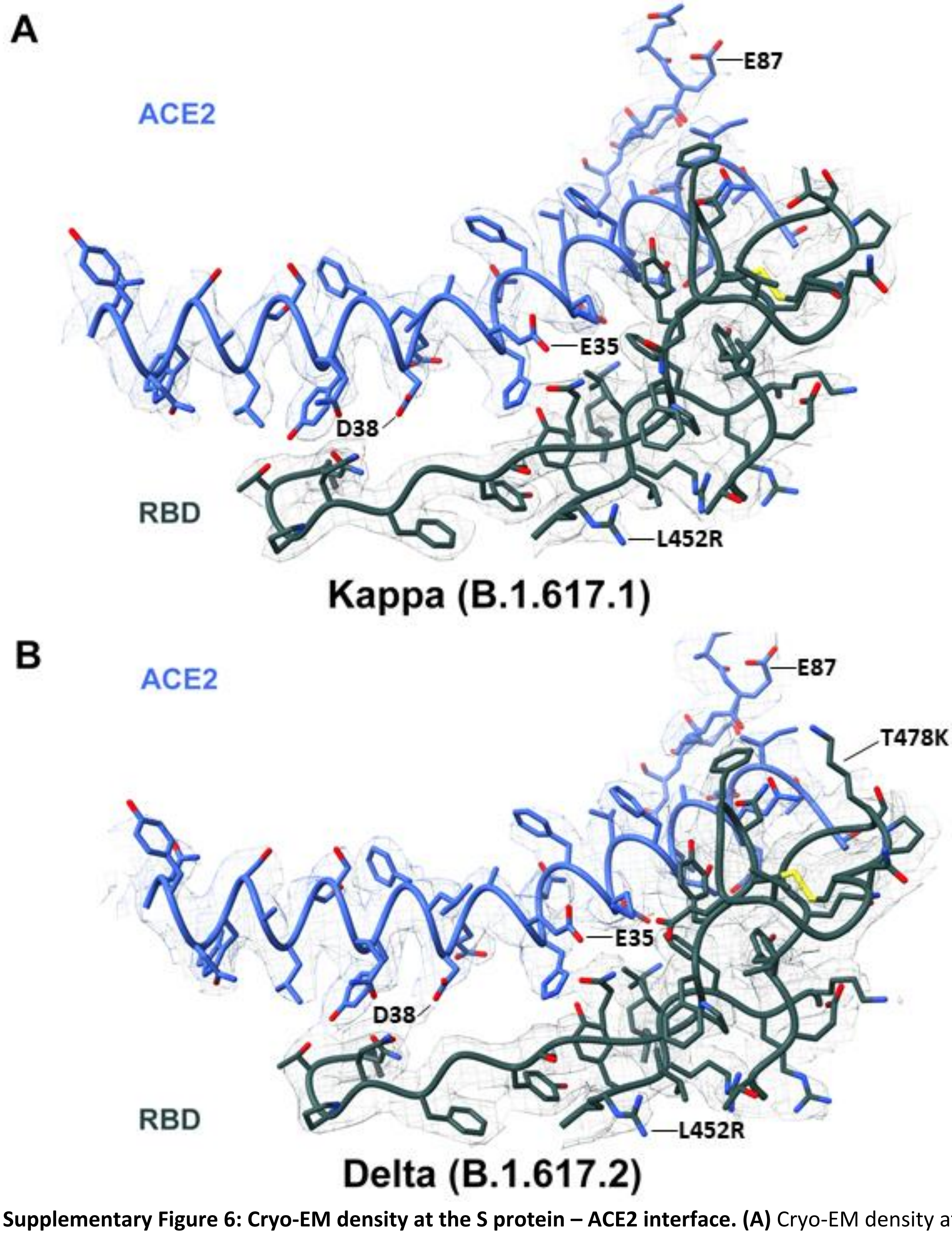

Supplementary Figure 6: Cryo-EM density at the S protein – ACE2 interface. (A) Cryo-EM density at the Kappa (B.1.617.1) variant RBD-ACE2 interface with discussed residues labelled. **(B)** As in (A) but for the Delta (B.1.617.2) variant.

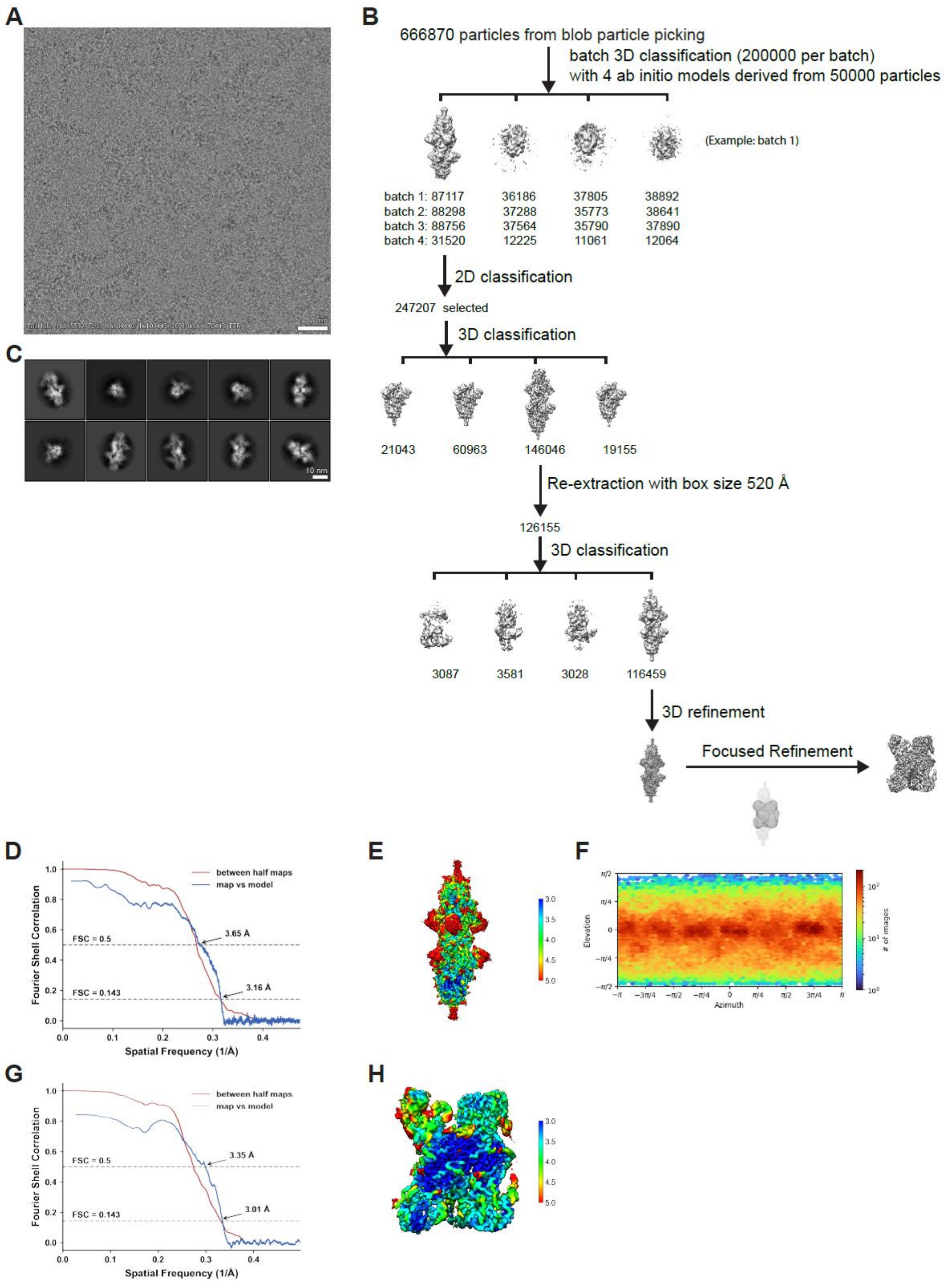

Supplementary Figure 7. Cryo-EM data processing and validation for the Kappa (B.1.617.1) spike protein ectodomain. (A) Representative cryo-EM micrograph from a total of 4486 micrographs. **(B)** Workflow of cryo-EM image processing. **(C)** Representative 2D classes. **(D)** Fourier Shall Correlation (FSC) curves for the global refinement. **(E)** Local resolution map for the global refinement. **(F)** Viewing direction distribution plot. **(G)** Fourier Shall Correlation (FSC) curves for the focused refinement of the dimer interface. **(H)** Local resolution map for the focused refinement.

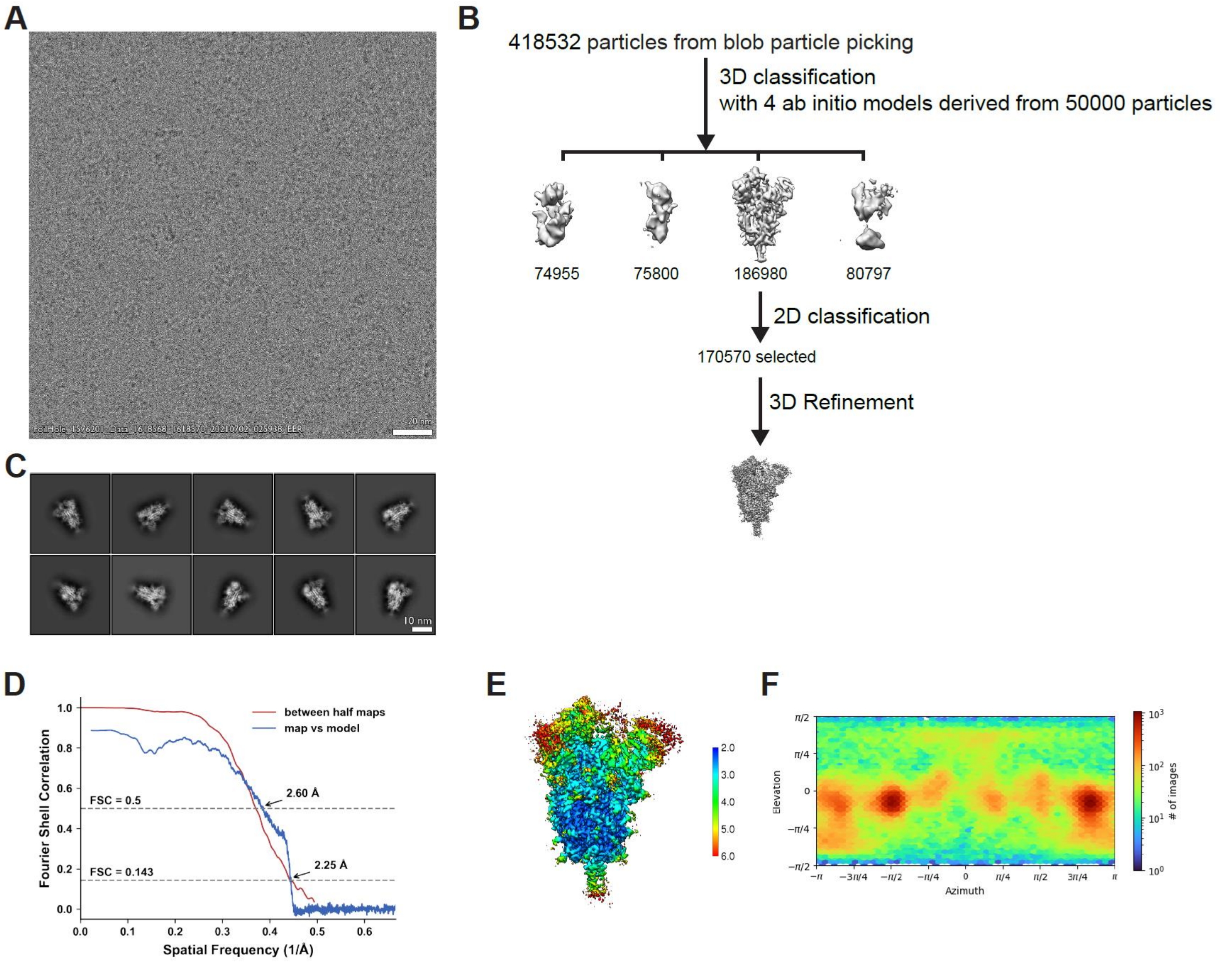

Supplementary Figure 8. Cryo-EM data processing and validation for the Delta (B.1.617.2) spike protein ectodomain. **(A)** Representative cryo-EM micrograph from 4860 micrographs. **(B)** Workflow of cryo-EM image processing. **(C)** Representative 2D classes. **(D)** Fourier Shall Correlation (FSC) curves for the global refinement. **(E)** Local resolution map for the global refinement. **(F)** Viewing direction distribution plot.

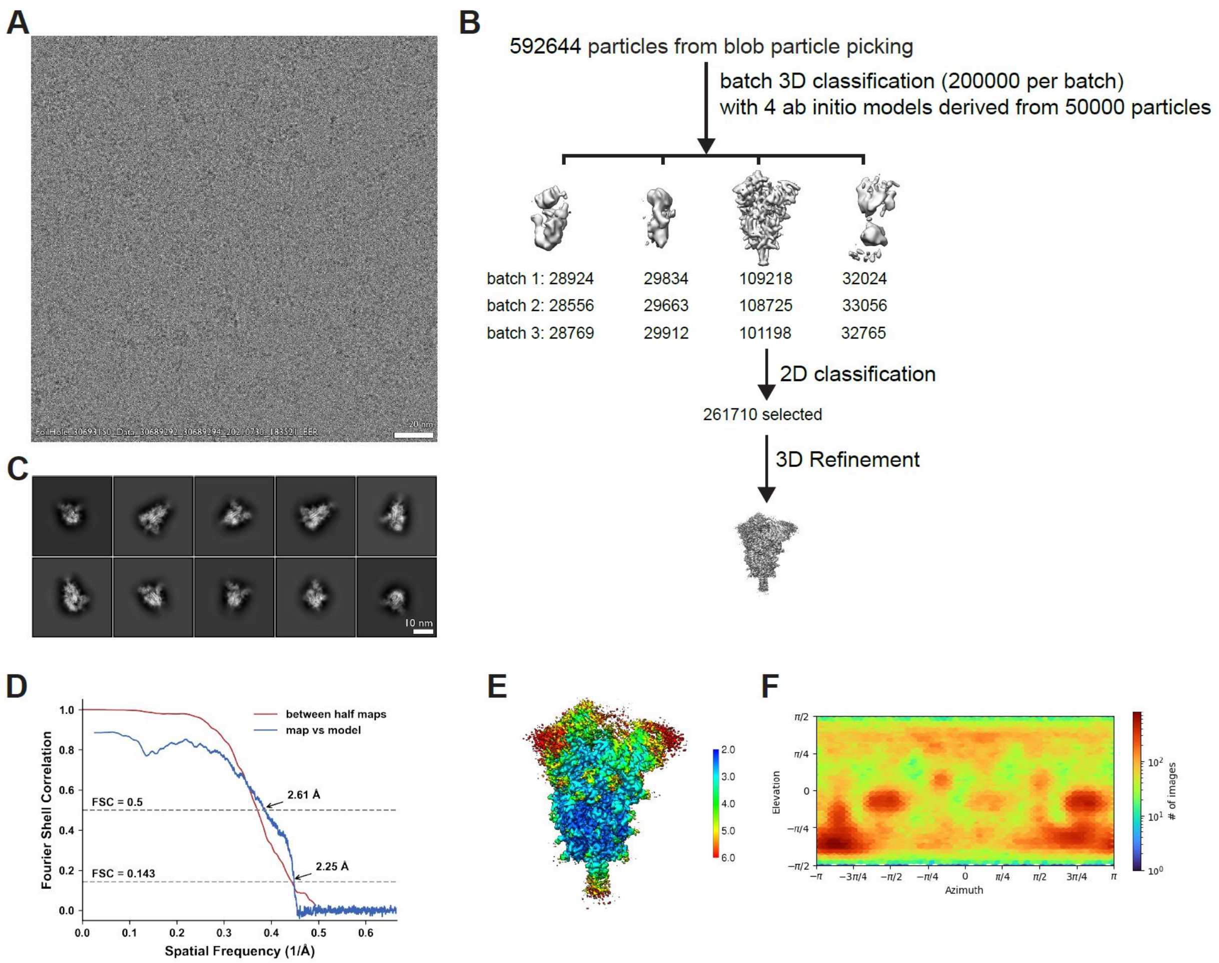

Supplementary Figure 9. Cryo-EM data processing and validation for the Kappa (B.1.617.1) + Q484A spike protein ectodomain. (A) Representative cryo-EM micrograph from 6750 micrographs. **(B)** Workflow of cryo-EM image processing. **(C)** Representative 2D classes. **(D)** Fourier Shall Correlation (FSC) curves for the global refinement. **(E)** Local resolution map for the global refinement. **(F)** Viewing direction distribution plot.

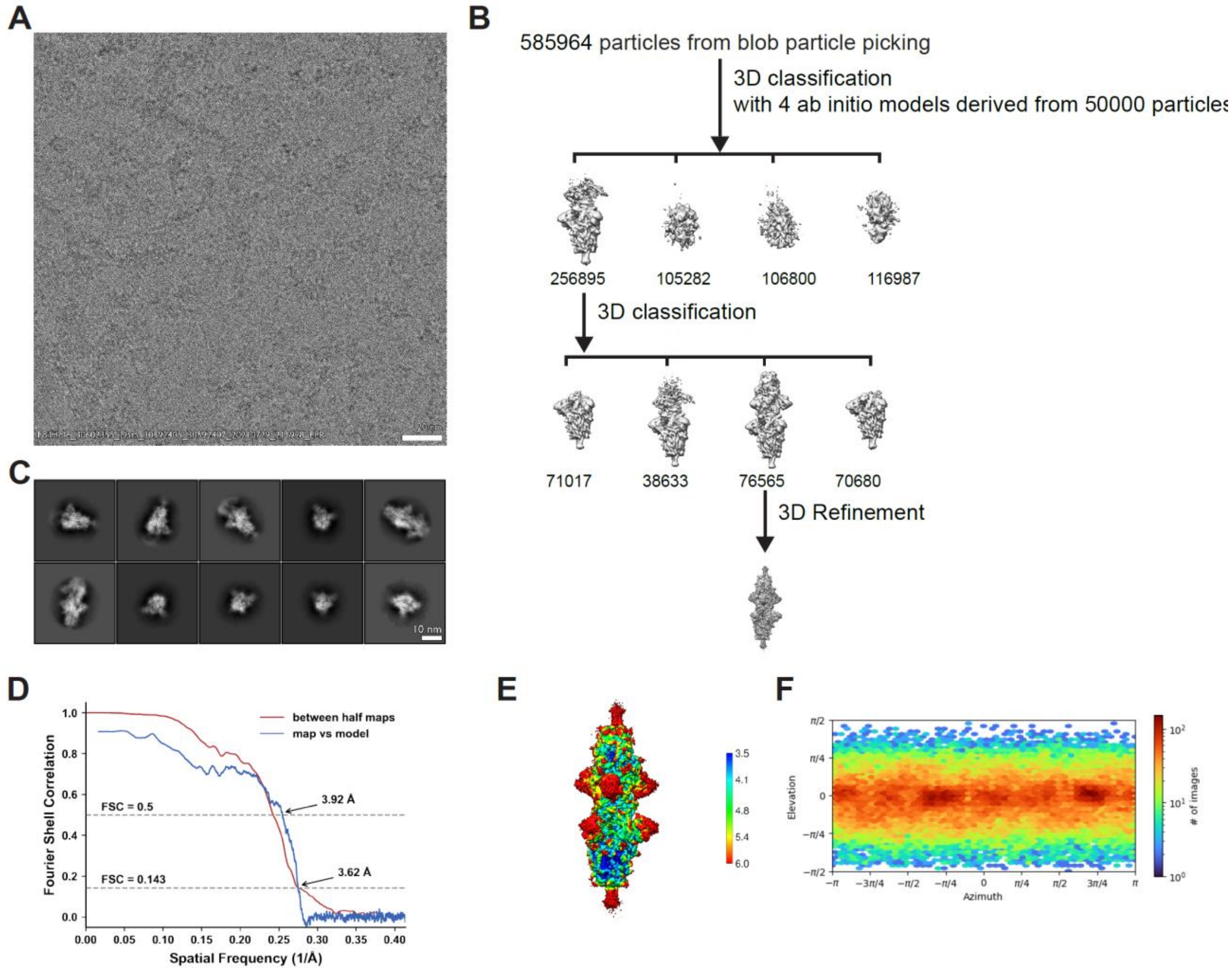

Supplementary Figure 10. Cryo-EM data processing and validation for the Kappa (B.1.617.1) + Q484I spike protein ectodomain. (A) Representative cryo-EM micrograph from 8380 micrographs. **(B)** Workflow of cryo-EM image processing. **(C)** Representative 2D classes. **(D)** Fourier Shall Correlation (FSC) curves for the global refinement. **(E)** Local resolution map for the global refinement. **(F)** Viewing direction distribution plot.

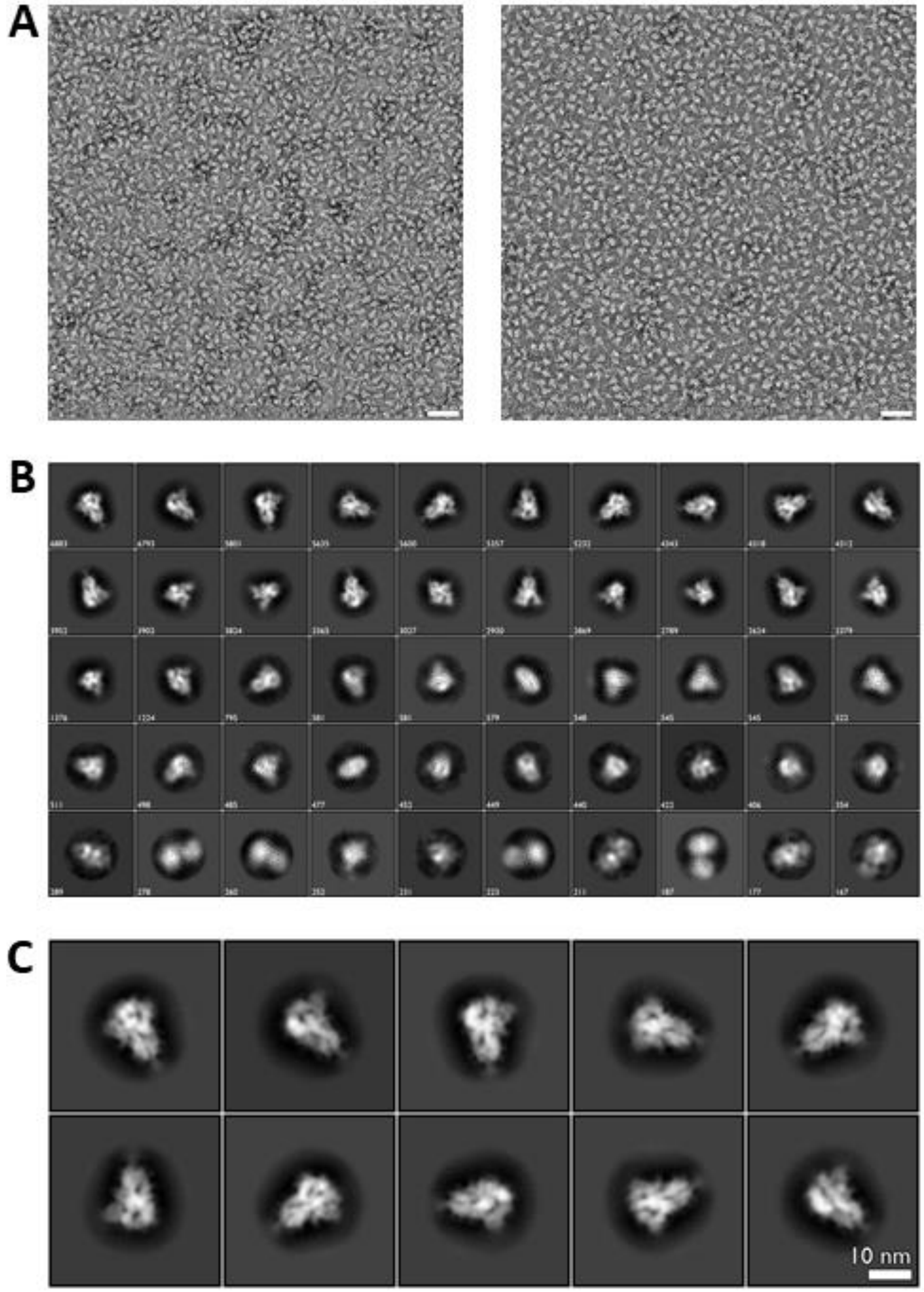

Supplementary Figure 11: The Kappa (B.1.617.1) variant S protein ectodomain reveals no dimerization under negative stain electron microscopy conditions. (A) Two representative micrographs selected from the total dataset (1467 micrographs) for the Kappa (B.1.617.1) variant S protein ectodomain. **(B)** All 2D class averages derived from the micrographs as represented in (A). **(C)** The ten most highly populated 2D class averages from (B).

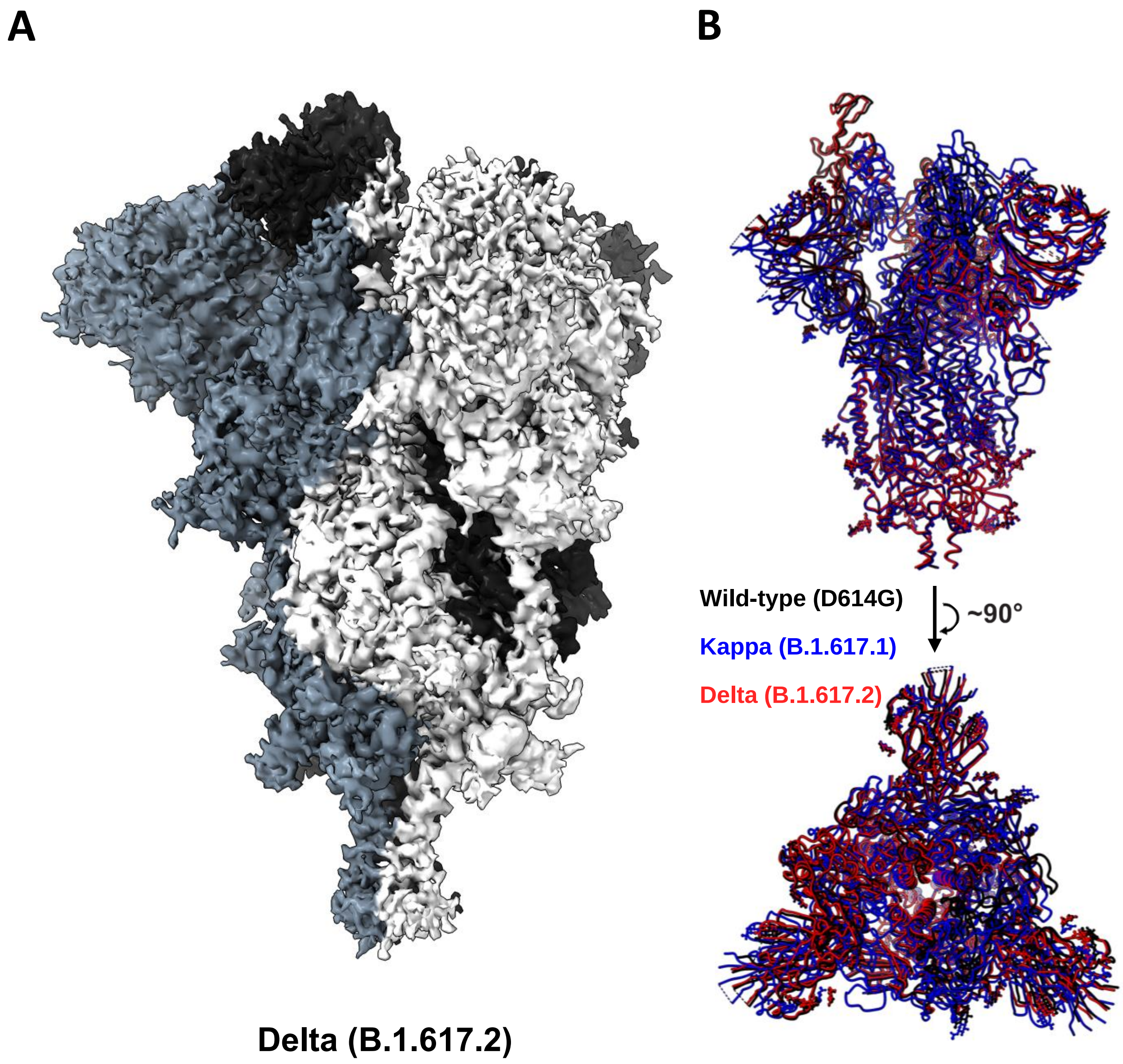

Supplementary Figure 12. CryoEm structure of the Delta (B.1.617.2) S protein ectodomain and its superposition with wild-type (D614G) and Kappa (B.1.617.1) structures. (A) Cryo-EM density map of the Delta (B.1.617.2) variant S protein with each protomer coloured in greyscale. **(B)** Superposition of the wild-type (D614G – Black), Kappa (B.1.617.1 – Blue), and Delta (B.1.617.2 – Red) atomic models.

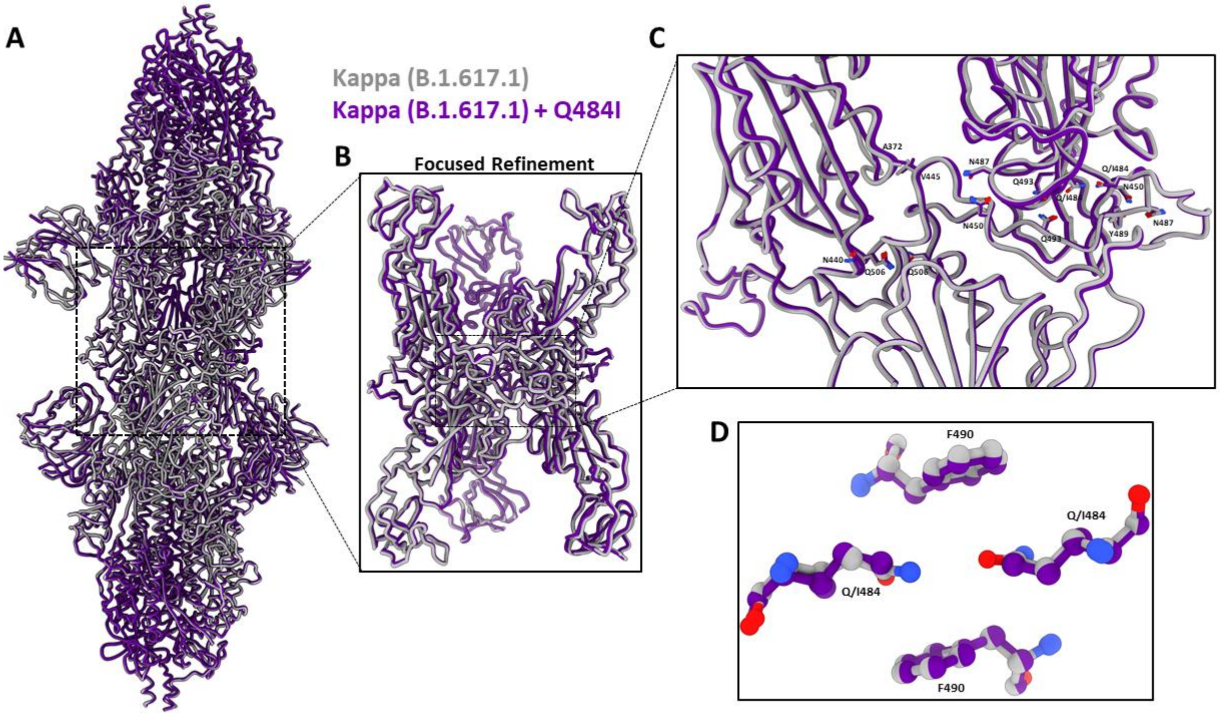

Supplementary Figure 13: Structural comparison of the Kappa and Kappa + Q484I dimer-of-trimers. (A) Global superposition of Kappa (grey) and Kappa + Q484I (purple) dimer-of-trimer atomic models. **(B)** As in (A) but for the focus-refined dimerization interface. **(C)** Detailed view of the dimerization interface as shown in Figure 4 with the discussed residues labelled. **(D)** A further focused view of the F490, Q484 and I484 residues at the 484-484 interaction site.

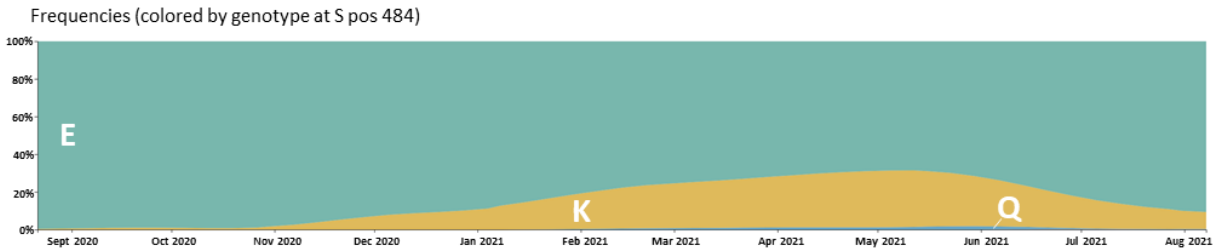

Supplementary Figure 14. Amino acid frequency at position 484 in global sequence deposits for the SARS-CoV-2 spike protein. Residue frequency was derived from the Global Initiative on Sharing Avian Influenza Data (GISAID) database between September 2020 and August 2021.

A

Sample	Sample Status	Vaccine Dose	Immunization to Serum Draw Time
P0	Vaccine post-COVID19	1st	4 Weeks
P1	Vaccine	1st	3 Weeks
P3	Vaccine	1st	6 Weeks
P5	Vaccine	1st	1 Week
P6	Vaccine	1st	3 Weeks
P8	Vaccine post-COVID19	1st	9 Weeks
P9	Vaccine post-COVID19	1st	7.5 Weeks
P10	Vaccine post-COVID19	1st	6.5 Weeks
P11	Vaccine pre-COVID19	1st	7 Weeks
P12	Vaccine post-COVID19	1st	8.5 Weeks
P13	COVID19	n/a	n/a (2 Weeks post-infection)
P14	Vaccine	2nd	4 Weeks

B

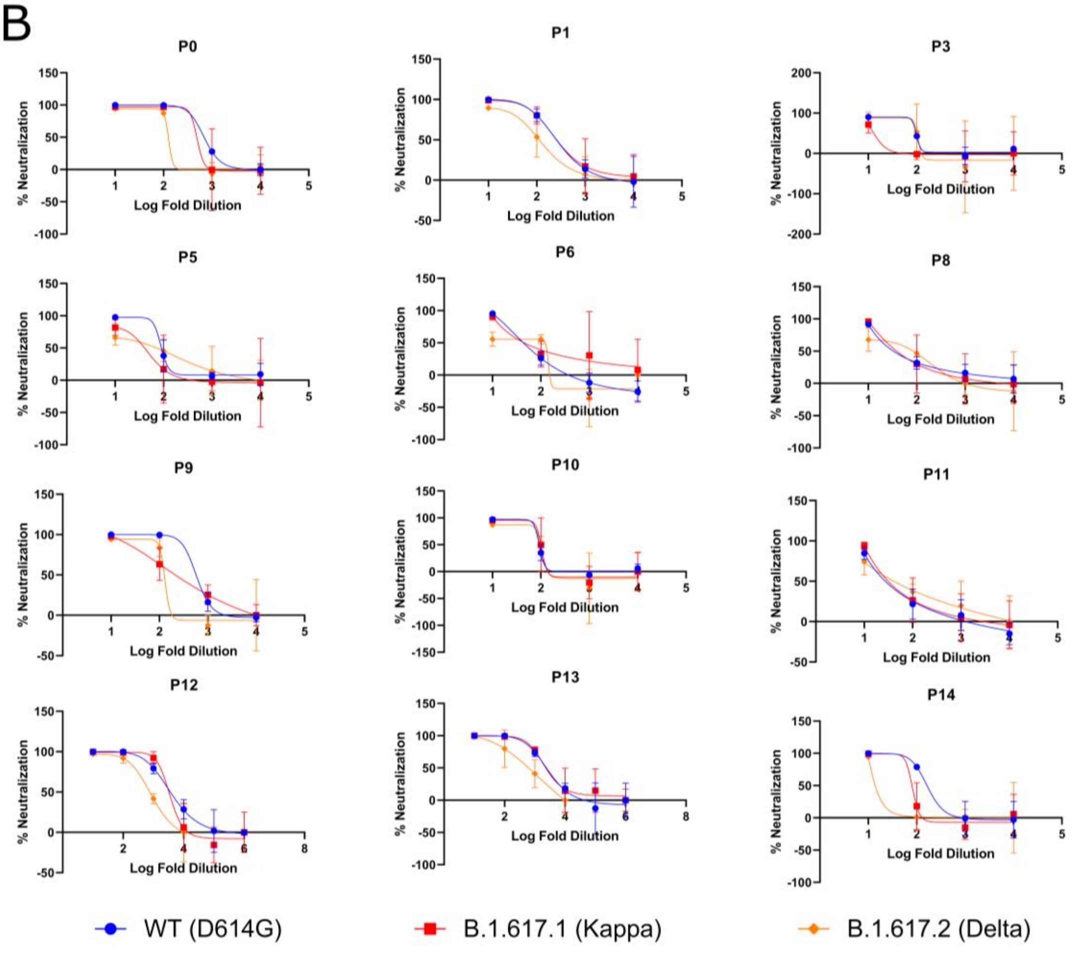

Supplementary Figure 15. Patient-derived sera sample information and raw pseudovirus neutralization data. **(A)** Patient-derived sera sample information including sample number, vaccination status, vaccination dose number and immunization to serum draw time. **(B)** Raw data for the neutralization of Wild-type (D614G), Kappa (B.1.617.1), and Delta (B.1.617.2) S protein pseudotyped viruses with patient-derived sera samples. Experiments were performed at least in technical duplicates, and the average values are shown. Error bars denote standard deviation. Source data are provided as a Source Data file.

Supplementary Table 1: Cryo-EM data collection, processing, refinement, and validation parameters for the structures reported in this publication.

Structure:	B.1.617.1		B.1.617.1(Q484A)	B.1.617.1(Q484I)		B.1.617.1 + ACE2		B.1.617.2	B.1.617.2 + ACE2	
	global refinement	focus refinement		global refinement	focus refinement	global refinement	focus refinement		global refinement	focus refinement
	(EMDB 25862)	(EMDB 25861)	(EMDB 25860)	(EMDB 25859)	(EMDB 25858)	(EMDB 25857)	(EMDB 25856)	(EMDB 25855)	(EMDB 25854)	(EMDB 25853)
	(PDB 7TF5)	(PDB 7TF4)	(PDB 7TF3)	(PDB 7TF2)	(PDB 7TF1)	(PDB 7TF0)	(PDB 7TEZ)	(PDB 7TEY)	(PDB 7TEX)	(PDB 7TEW)
**Data collection**										
Microscope	Titan Krios G4		Titan Krios G4	Titan Krios G4		Titan Krios G4		Titan Krios G4	Titan Krios G4	
Detector	Falcon4		Falcon4	Falcon4		Falcon4		Falcon4	Falcon4	
Voltage (kV)	300		300	300		300		300	300	
Nominal magnification	155,000		155,000	155,000		155,000		155,000	155,000	
Defocus range (μm)	-3.0 to -0.5		-3.0 to -0.5	-3.0 to -0.5		-3.0 to -0.5		-3.0 to -0.5	-3.0 to -0.5	
Physical pixel (Å)	0.5		0.5	0.5		0.5		0.5	0.5	
Electron dose ($e^-/Å^2$)	40		40	40		40		40	40	
Exposure rate ($e^-/Å^2$/sec)	24		24	24		24		24	24	
Format of movies	EER		EER	EER		EER		EER	EER	
Number of raw frames	399		399	399		399		399	399	
Number of movies	8,907		6,750	8,380		8,478		4,860	9,630	
**Data processing**										
Number of fractions	40		40	40		40		40	40	
Number of extracted particles	666,870		592,644	585,964		619,611		418,532	804,096	
Number of particles for final map	116,459		261,710	76,565		105,037		170,570	97,827	
Symmetry imposed	C1	C1	C1	C1	C1	C1	C1	C1	C1	C1
Resolution (Å)	3.16	3.01	2.25	3.62	3.57	3.02	3.27	2.25	3.27	3.52
FSC threshold	0.143	0.143	0.143	0.143	0.143	0.143	0.143	0.143	0.143	0.143
**Refinement**										
Initial model used	7MJG	7MJG	7MJG	7MJG	7MJG	7MJM	7MJN	7MJG	7MJM	7MJN
Map sharpening B-factor ($Å^2$)	53.3	53.7	43.4	37.6	64.5	50.1	76.6	35.1	42.9	94.0
Composition (#)										
Atoms	50,298	12,792	21,873	50,292	12,786	26,849	6,553	21,867	26,839	6,555
Residues	6,228	1,608	2,703	6,228	1,608	3,303	797	2,703	3,303	797
Ligands	NAG:120	NAG:12	NAG:56	NAG:120	NAG:12	NAG:62	NAG:7	NAG:56	NAG:62	NAG:7
B-factor ($Å^2$)										
Protein (min/max/mean)	50.19/261.43/134.79	44.03/149.98/80.04	38.78/214.65/96.20	66.75/282.21/164.45	74.37/230.07/124.54	56.74/325.77/150.64	76.49/206.75/115.23	37.32/238.01/101.10	63.25/561.65/188.59	101.09/244.57/156.07
Ligand (min/max/mean)	100.53/283.60/168.86	76.17/133.82/96.25	59.93/218.08/109.14	121.54/311.42/201.73	124.60/183.36/149.78	89.65/285.93/153.23	116.95/149.58/132.83	56.12/241.40/114.62	95.97/566.98/180.20	164.13/210.83/180.46
Bonds (RMSD)										
Length (Å) (# > 4σ)	0.003 (0)	0.005 (0)	0.004 (0)	0.003 (0)	0.004 (0)	0.004 (0)	0.005 (0)	0.004 (0)	0.004 (0)	0.005 (0)
Angles (°) (# > 4σ)	0.706 (15)	0.835 (4)	0.787 (6)	0.705 (14)	0.753 (2)	0.780 (8)	0.885 (1)	0.782 (6)	0.767 (7)	0.840 (1)
CC_mask	0.83	0.82	0.83	0.82	0.81	0.82	0.84	0.83	0.83	0.82
**Validation**										
Ramachandran plot										
Residues favored (%)	97.90	97.18	98.00	97.88	97.49	98.52	98.49	98.00	98.55	98.61
Residues disallowed (%)	0.00	0.00	0.00	0.00	0.00	0.03	0.00	0.00	0.00	0.00
Rotamer outliers (%)	0.37	0.00	0.00	0.35	0.36	0.10	0.71	0.00	0.17	0.14
Clash score	3.03	3.59	3.03	3.32	2.67	3.12	2.42	3.31	2.89	2.81
MolProbity score	1.12	1.30	1.09	1.15	1.16	1.10	1.02	1.12	1.08	1.07

Supplementary Table 2: Kits and reagents related to the methods section.

Pierce 1-Step Ultra Substrate Solution	Thermo Fisher Scientific	Cat. # 34028
Q5 Site-Directed mutagenesis kit	NEB	Cat. # E0554S
Lenti-X™ GoStix™ Plus	Takara Bio	Cat. # 631281
ONE-Glo™ EX Luciferase Assay System	Promega	Cat. # E8130

Supplementary Table 3: Antibodies related to the methods section.

Antibody	Reference	Catalogue Number
VH ab8	[17, 18]	N/A
IgG1 ab1	[17, 18]	N/A
Fab S309	[22]	N/A
Fab S2M11	[21]	N/A
Fab 4A8	[19]	N/A
Fab 4-8	[20]	N/A
goat anti-human IgG	Jackson ImmunoResearch	Cat. # 109-035-088

Supplementary Table 4: Recombinant proteins related to the methods section.

Ace2 (18-615)	New England Biolabs	Cat. # 73775S

Supplementary Table 5: Cell lines related to the methods section.

Expi293F	Thermo Fisher Scientific	Cat# A14527

Supplementary Table 6: Recombinant DNA and Oligonucleotides related to the methods section.

pαH SARS-CoV-2 S HexaPro	[46]	Addgene #154754
pαH HexaPro D614G	[23]	N/A
pcDNA3.1 Fab S309 Light Chain	[23]	N/A
pcDNA3.1 Fab S309 Heavy Chain	[23]	N/A
pcDNA3.1 Fab S2M11 Light Chain	[23]	N/A
pcDNA3.1 Fab S2M11 Heavy Chain	[23]	N/A
pcDNA3.1 Fab 4A8 Light Chain	This paper	N/A
pcDNA3.1 Fab 4A8 Heavy Chain	This paper	N/A
pcDNA3.1 Fab 4-8 Light Chain	This paper	N/A
pcDNA3.1 Fab 4-8 Heavy Chain	This paper	N/A

Supplementary Table 7: Software and Algorithms related to the methods section.

GraphPad Prism	GraphPad 7.0	https://www.graphpad.com/scientific-software/prism/
EPU 2 automated acquisition	Thermo Fisher Scientific	https://www.thermofisher.com/us/en/home/electron-microscopy/products/software-em-3d-vis/epu-software.html
UCSF Chimera	[47]	https://www.cgl.ucsf.edu/chimera/
UCSF ChimeraX (v.1.1.1)	[57]	https://www.cgl.ucsf.edu/chimerax/
RELION 3.1	[48]	https://github.com/3dem/relion/releases/tag/3.1.0
crYOLO (v1.7.4)	[49]	https://pypi.org/project/cryolo/
cryoSPARC live (v3.2)	[50]	https://cryosparc.com/live
COOT (v.0.9.3)	[54]	https://www2.mrc-lmb.cam.ac.uk/personal/pemsley/coot/
Phenix (v.1.19)	[55]	https://phenix-online.org/
MolProbity (v.4.5.1)	[56]	http://molprobity.biochem.duke.edu/
